# New Genes Interacted With Recent Whole-Genome Duplicates in the Fast Stem Growth of Bamboos

**DOI:** 10.1093/molbev/msab288

**Published:** 2021-09-28

**Authors:** Guihua Jin, Peng-Fei Ma, Xiaopei Wu, Lianfeng Gu, Manyuan Long, Chengjun Zhang, De-Zhu Li

**Affiliations:** 1 Germplasm Bank of Wild Species, Kunming Institute of Botany, Chinese Academy of Sciences, Kunming, Yunnan, China; 2 Basic Forestry and Proteomics Research Center, College of Forestry, Fujian Agriculture and Forestry University, Fuzhou, Fujian, China; 3 Department of Ecology and Evolution, The University of Chicago, Chicago, IL, USA

**Keywords:** fast stem growth, orphan genes, de novo genes, WGD, woody bamboos, evolutionary innovation

## Abstract

As drivers of evolutionary innovations, new genes allow organisms to explore new niches. However, clear examples of this process remain scarce. Bamboos, the unique grass lineage diversifying into the forest, have evolved with a key innovation of fast growth of woody stem, reaching up to 1 m/day. Here, we identify 1,622 bamboo-specific orphan genes that appeared in recent 46 million years, and 19 of them evolved from noncoding ancestral sequences with entire de novo origination process reconstructed. The new genes evolved gradually in exon−intron structure, protein length, expression specificity, and evolutionary constraint. These new genes, whether or not from de novo origination, are dominantly expressed in the rapidly developing shoots, and make transcriptomes of shoots the youngest among various bamboo tissues, rather than reproductive tissue in other plants. Additionally, the particularity of bamboo shoots has also been shaped by recent whole-genome duplicates (WGDs), which evolved divergent expression patterns from ancestral states. New genes and WGDs have been evolutionarily recruited into coexpression networks to underline fast-growing trait of bamboo shoot. Our study highlights the importance of interactions between new genes and genome duplicates in generating morphological innovation.

## Introduction

Evolutionary innovations are throughout the Tree of Life and contribute to organismal diversification (Pigliucci 2008; Wagner and Lynch 2010). The morphological and physiological innovations allow organisms to explore new niches to generate biological diversity. Examples of major innovations have been known from flowers in angiosperms, lung in tetrapods, feathers in birds, and wings in insects (Liem 1988; Averof and Cohen 1997; Albert et al. 2002; Prum and Brush 2002). How novel traits originate becomes a fundamental question in evolutionary biology. However, the majority of studies have focused on external environmental induce for innovations. We know little about the genetic basis underlying the appearance of phenotypic novelties, despite a link between the innovations and genomic novelty in general and new genes in particular that has long been hypothesized (Chen et al. 2013; Erwin 2021). Recent studies provided direct evidence in support of this hypothesis by attributing phenotypic innovation to the evolution of new genes (Kaessmann 2010; Chen et al. 2011; Long et al. 2013; Erwin 2015; Santos et al. 2017). Evolutionary new genes, also called as lineage-specific genes that emerged recently in a given lineage, can be derived from existing genetic elements like the process of divergence of duplicated genes (Long et al. 2013). A dramatic genetic novelty is the orphan genes created through various molecular evolutionary processes, especially de novo genes from noncoding sequences with several well-documented organisms (Carvunis et al. 2012; McLysaght and Guerzoni 2015; Xie et al. 2019; Zhang et al. 2019; Heames et al. 2020). New genes in animals and plants tend to be preferentially expressed in the male reproductive tissues (Dai et al. 2006), and thus an “out of testis” hypothesis for the emergence of new genes has been proposed (Dai et al. 2006; Vinckenbosch et al. 2006).

Recent advances in genome sequencing of multiple related species enable to explore the relationship between evolutionary innovations and origin of new genes. The species distribution of a gene suggests its possible age (Long et al. 2013). Computationally, by studying genome evolution within a phylogenetic content, phylostratigraphy can detect evolutionary origin of orphan genes through sequence similarity searches in genomes across the Tree of Life (Domazet-Loso et al. 2007). This approach identifies specifically the origin of new genes which lack traceable homologs to existing genes in other lineages. These orphan genes may have been created in several alternative mechanisms from rapid sequence evolution of homologous copies to lateral transfer to de novo origination (Moyers and Zhang 2016; Domazet-Loso et al. 2017; Zhang et al. 2019; Vakirlis et al. 2020; Jin et al. 2021). It assigns every gene within a genome to a given phylogenetic rank designated as phylostratum (PS)–describing the age of gene in a phylogenetic context, parallel to chronostratigraphic age in geology. The genes underlying innovation are thus hypothesized to be enriched in the corresponding phylostratum when innovation first emerged (Sestak et al. 2013; Sestak and Domazet-Loso 2015; Trigos et al. 2017; Shi et al. 2020). To distinguish the orphan genes that are created de novo from other alternative mechanisms, the closely related species is searched for their noncoding ancestral sequences and reconstruction of origination processes (Murphy and McLysaght 2012; Zhang et al. 2019). In combination with gene expression analyses, further insights can be gained into the birth process of new genes and their potential functions. Two similar transcriptome indices for gene, the transcriptome age index (TAI) and transcriptome divergence index (TDI), have been developed with higher values indicating younger and more divergent transcriptome (Domazet-Loso and Tautz 2010; Quint et al. 2012). Moreover, new genes need to be recruited into the genetic network to be functional and the interaction of genes can be identified by the gene coexpression network based on common expression profiles (Shao et al. 2019).

**Fig. 1.**
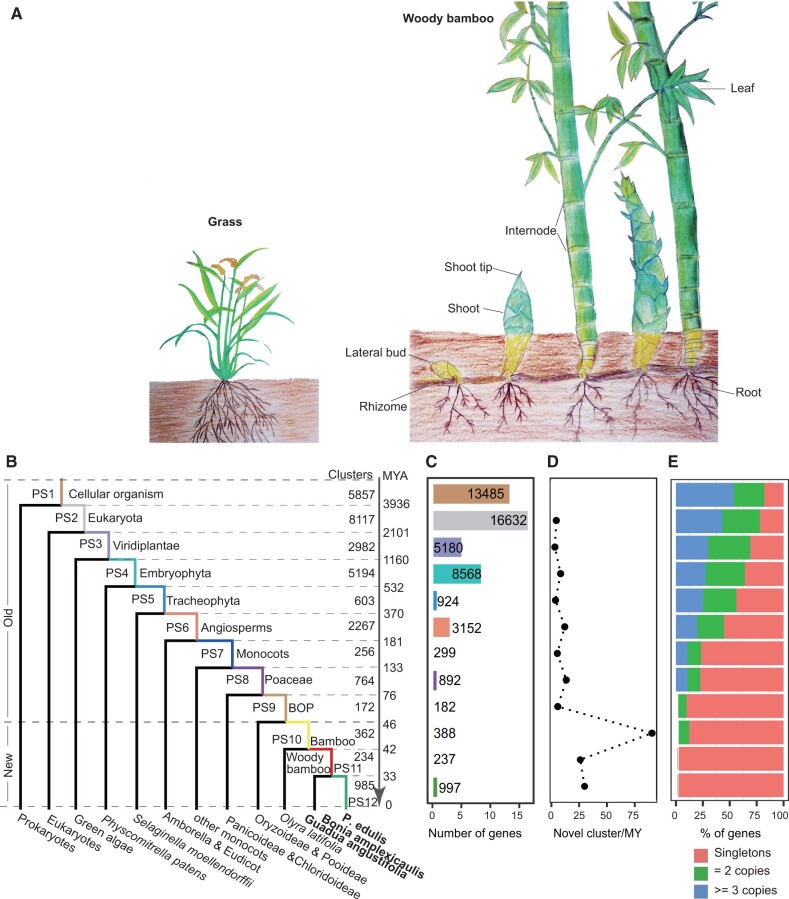
Morphological character and phylostratigraphic age of *Phyllostachys edulis*. (*A*) Comparison of the growth pattern of grass and woody bamboo. (*B*) Phylostratigraphic ages distribution of *P. edulis*. Numbers denote the number of nonredundant gene clusters per phylostratum (PS1−PS12). The tree stratifies species by major evolutionary innovations, from the emergence of simple unicellular organisms up to *P. edulis.* The dating of phylogenetic tree is from TimeTree (Kumar et al. 2017). (*C*) Number of genes per phylostratum. (*D*) Gene fixation rate per PS. (*E*) Gene copies distribution of each cluster.

Here we use the bamboos (Poaceae, Bambusoideae) as a model system to study this question. Bambusoideae is the only major grass lineage diversifying into the forest habit with more than 1,600 species worldwide (Soreng et al. 2017). In contrast to other grasses, the majority of bamboo species have woody, tall, and lignified stems (fig. 1*A*), reaching up to approximately 20 m in the widely cultivated moso bamboo (*Phyllostachys edulis*) and even more than 30 m in a few species such as *Dendrocalamus sinicus* (Chang and Wu 2000; Song et al. 2016), being the tallest grasses in the world. Furthermore, the growth and development of these tall stems can be rapidly completed within 2 − 3 months, showing a “slow-fast-slow” pattern (Zhou 1983). For example, the shoot of moso bamboo can grow 1 m in height within 24 h (Ueda 1960; Liese and Kohl 2015; Song et al. 2016), which is hundreds of times faster than other woody trees (Li et al. 2020). This trait of fast growth of woody stem could be considered as a key innovation in bamboos, likely facilitating their adaptation to the forest habit with access to light and thus vast species diversification. Another unique trait of bamboos is the infrequent flowering with intervals as long as 20−60 years while a high ability of propagation by clone (Janzen 1976).

Previous works have mainly focused on morphology, anatomy, and physiology to study the trait of fast growth of woody stem in bamboos (Ueda 1960; He et al. 2002; Wang, Ren, et al. 2012; Song et al. 2016). Only a few genes related to plant hormones network, cell cycle regulation and cell wall metabolism have been investigated for the evolution of this unique trait (Cui et al. 2012; Peng, Lu, et al. 2013; Li et al. 2018; Wei et al. 2018). Recent genomic studies revealed the polyploidization history of woody bamboos (Guo et al. 2019), which was suggested to be related to the origin and evolution of this trait. By taking advantage of bamboo genomes recently sequenced, we combined phylostratigraphy with a noncoding ancestral search for orphan and de novo gene candidates (Zhang et al. 2019), to determine the phylogenetic age of genes in the genomes of woody bamboos and found that new genes were highly and specifically expressed in the shoot tissue. The noncoding ancestral sequences, as core evidence of de novo genes, are rarely detected in metazoans from mammals to invertebrates. The *Oryza* genomes in the grass family provided the first genomic evidence for existence of de novo genes (Zhang et al. 2019). We inferred that genomes of bamboos, as a grass subfamily Bambusoideae, likely contain de novo genes with detectable noncoding ancestral sequences. Our study highlights a central role for orphan and de novo genes, through interaction with whole-genome duplicates (WGDs), in the origination and evolution of morphological innovations in bamboos, pinpointing a general correlation between phenotypic innovations and genomic novelty.

## Results

### Phylogenetic Origination of Woody Bamboo Genes and Their Features

To trace the phylogenetic origins of woody bamboo genes, we selected one herbaceous bamboo *Olyra latifolia* and three woody bamboos with sequenced genomes (Zhao et al. 2018; Guo et al. 2019) for phylostratigraphic analysis, together with other 65 representative genomes across the Tree of Life (supplementary table 1, Supplementary Material online). The three woody species, *Bonia amplexicaulis*, *Guadua angustifolia*, and *P. edulis*, represent all of the three major lineages of woody bamboos in the Bambusoideae (Guo et al. 2019). We separately adopted the genes of *Bo. amplexicaulis and P. edulis*, for which the quality of genome assemblies is much higher than that of *G*. *angustifolia*, as query to conduct analyses. In considering the polyploid nature of woody bamboos, the paralogous genes within genome were gathered to generate nonredundant queries (supplementary fig. 1, Supplementary Material online). In all, we defined 12 phylostrata (PS) ranks ranging from the oldest PS1 (cellular organisms) to the youngest PS12 (species specific) based on the ladder-like phylogenetic tree and nearly identical results were obtained in using *P. edulis and Bo. amplexicaulis* (fig. 1*B*−*E*; supplementary fig. 2, Supplementary Material online). We thus focused on the results below using the genes of *P. edulis* as query.

A total of 50,936 genes from *P. edulis* were assigned to 12 PSs (fig. 1*B and C*) and the number of genes per rank was positively correlated with gene ages (Pearson correlation coefficient *r* = 0.7742, *P* = 0.00312). There were five peaks of appearance of genes during the evolutionary history of bamboos, which were associated with the emergence of cellular organisms (PS1), Eukaryota (PS2), Viridiplantae (PS3), Embryophyta (PS4), and Angiosperms (PS6). This pattern of distribution for gene ages was also observed in the previous studies of *Arabidopsis* and rice (*Oryza sativa*) (Cui et al. 2015). An overwhelming proportion of 96.82% genes could be traced back to the old gene ranks from PS1 to PS9 before the origin of bamboos. As such, there were only 1,622 genes emerging in the bamboo lineage, including 388 of PS10, 237 of PS11, and 997 of PS12 (supplementary table 2, Supplementary Material online), which could be considered as bamboo-lineage specific. We called them evolutionary new genes, or orphan genes, thereafter. Splitting from the subfamily Pooideae approximately 46 million years (Mys) ago, the bamboo lineage has a rate of origination of approximately 35 orphan genes/My. We also calculated the fixation rate of gene families for different PSs and found that the internode leading to bamboos corresponding to PS10 had the highest rate of 90.5 clusters/My (fig. 1*D*;supplementary table 3, Supplementary Material online). Interestingly, the opportunity for genes to being singleton decreased with their ages, and 41.15% of the PS1 genes had multiple copies whereas 98.78% of the PS12 genes were all single copy (fig. 1*E*).

### New Genes Preferentially Expressed in the Fast-Growing Phase of Shoot

**Fig. 2.**
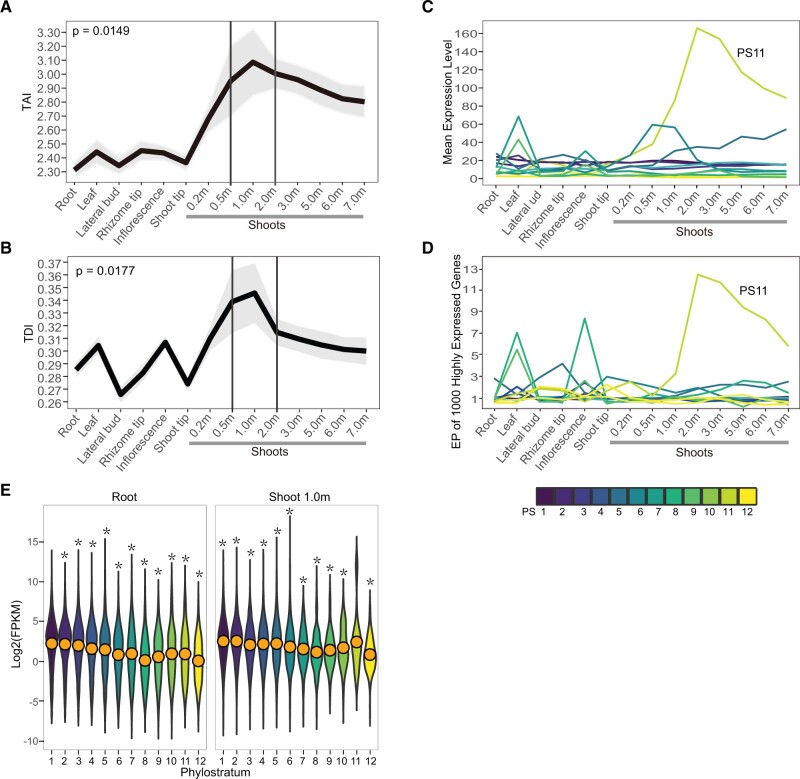
Evolutionary transcriptome profiles of *Phyllostachys edulis*. *(A)* TAI profiles. A high TAI value indicates that the sample expresses a high number of younger genes. (*B*) TDI profile. The gray shade represents the standard deviation by permutation analysis. The *P* value was derived by application of flat line test. (*C*) Mean expression level of genes from each phylostratum (PS). (*D*) EP of the top 1,000 highly expressed genes. (*E*) The expression level of each PS in root and in shoot 1.0 m. The Wilcoxon rank sum test was used for comparison of expression difference between PS1 and other PSs (*P < 0.05) in root, and between PS11 and other PSs in shoot 1.0 m.

To identify the expression pattern of each phylostratum, we used transcriptome data from seven tissues of *P. edulis*, including root, leaf, rhizome tip, later bud, shoot tip, inflorescence, and shoot (supplementary fig. 3 and table 4, Supplementary Material online). Among them, the shoot tissue contained eight different development stages according to the height of shoot (fig. 2*A*). Expression was observed for 81.10% of the total genes (41,307 genes) having a value of fragments per kilobase million (FPKM) ≥1 at least in one of the seven tissues (supplementary tables 5 and 6, Supplementary Material online). Surprisingly, the new genes tended to be more expressed than the old ones with 71.91% of PS10, 86.92% of PS11, and 83.65% of PS12 genes expressed whereas a range of 66.03−83.42% of PS1−PS9 genes expressed.

Phylotranscriptomic analyses combing gene age and expression information revealed generally higher TAI values for the shoot than the other tissues (fig. 2*A*). This value rose to the highest point in the shoots of 0.5−2.0 m in height, corresponding to the turning point from the slow to fast growth of the *P. edulis* shoot (Song et al. 2016). A similar trend was also observed with TDI (fig. 2*B*). These results suggested higher expression level of new genes and thus divergent transcriptomes in the shoot, particularly at the height of 0.5−2.0 m. Similarly, at the individual level of phylostratum, highly elevated expression level of new genes of PS11, originating at the common ancestor of woody bamboos, was found in the shoots and also peaking at the 2.0 m stage (fig. 2*C*). All the genes from the remaining phylostrata with the exception of PS5−PS7 and PS9, whose genes had a much lower degree of altered expression than that of PS11, showed no significant changes of expression across the tissues. To avoid the impact of potential spurious new genes with low expression levels, we further analyzed gene ages and expression levels for the top 1,000 highly expressed genes per tissue. An index of expression preference (EP = expression frequency/gene frequency) was calculated for each phylostratum (supplementary tables 7 and 8, Supplementary Material online) with greater value pointing to preferred expression of genes in a given tissue. Results showed that the genes of PS11 had the highest EP value in the shoot tissue (fig. 2*D*), further proving new genes highly and specifically expressed in the shoot. In addition, for the average gene expression level, the genes of PS1 had the highest expression level and significantly higher than other PSs in all the tissues excluding shoot (*P* < 0.05 in the Wilcoxon rank sum test, fig. 2*E*;supplementary fig. 4, Supplementary Material online). In contrast, the genes of PS11 showed the highest expression level in the shoot, indicating a special role of the PS11 genes within the new genes in the rapid growth of *P. edulis* shoot.

To check whether these findings were applicable to other woody bamboos, we conducted a similar analysis for the *D*. *sinicus* with available transcriptome data (Chen et al. 2018). In agreement with the observations in *P. edulis*, the TAI value reached a peak at the fast growth stage of the *D. sinicus* shoot and new genes were also highly expressed in this stage (supplementary fig. 5, Supplementary Material online). Together, these results suggested that the findings above could hold true for the whole lineages of woody bamboos. In sum, the expression data above reveal that the new genes especially those from PS11 have played an important role in the rapid growth of shoot for woody bamboos.

### Nineteen PS11 Genes Identified as De Novo Genes and Their Step-Wise Evolution of New Genes

**Fig. 3.**
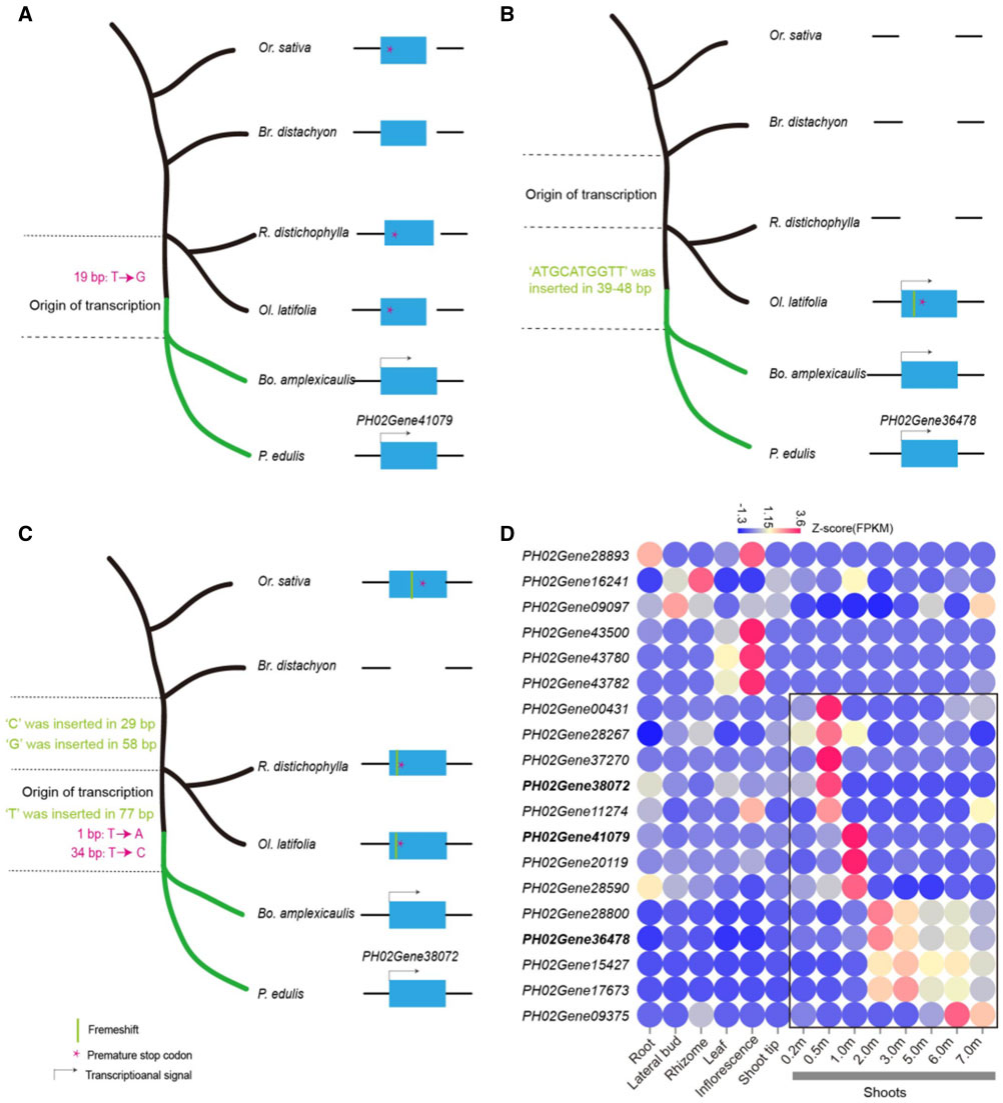
De novo gene birth of PS11 genes and their expression. (*A*) Origination process for the de novo gene *PH02Gene41079*. De novo creation of ORF can be one “T → G” substitute at 19 bp to remove the premature stop codon in the ancestor of woody bamboos. Its expression is detected in two woody bamboo species. The sequence alignments of *PH02Gene41079* were exhibited in supplementary figure 6, Supplementary Material online. (*B*) Origination process for the de novo gene *PH02Gene36478*. One 8-bp deletion resolved frameshift and the following premature stop codon. The noncoding ancestral sequence in *Ol. latifolia* acquired expression. The sequence alignments of *PH02Gene38478* were exhibited in supplementary figure 7, Supplementary Material online. The gap in the species represents the absence of homologous sequence from that species. (*C*) Origination process for the de novo gene *PH02Gene38072* via two-step process, as follows. 1) Two 1-bp frameshifts resolved the frameshift in *Or. sativa* and premature stop codon. 2) One “T to A” substitution created start codon, and “T to C” substitution resolved the premature stop codon, and one “T” insert resolved frameshift in *R. distichophylla and Ol. latifolia.* The sequence alignments of *PH02Gene38072* were exhibited in supplementary figure 8, Supplementary Material online. (*D*) Expression heatmap for 19 de novo genes. Black box highlighted shoot-biased expression pattern. The expressions of the three exampled de novo genes were shown in bold.

To gain insight into the origination process of new genes of PS11, we performed a pipeline as in Zhang et al. (2019) to detect those of being de novo origin, which could represent veritable leaps of evolutionary innovation (Blevins et al. 2021). Among the 237 PS11 genes, we identified 19 de novo genes with high confidence (supplementary table 9, Supplementary Material online). All of these de novo genes had complete coding frames in the woody bamboos *P. edulis and Bo. amplexicaulis*, whereas their orthologous sequences were noncoding in at least one of the four outgroup species (*Ol. latifolia*, *Raddia distichophylla*, *Brachypodium distachyon*, and rice). Three examples of identified genes were shown for the de novo origination processes in figure 3*A−**C*.

For the de novo gene *PH02Gene41079*, it became a gene through one nucleotide substitution at 19 bp from “TAG” to “GAG” to remove premature stop codon (supplementary fig. 6, Supplementary Material online). For *PH02Gene36478*, it originated by an 10 bp deletion to resolve frameshift and the premature stop codon (fig. 3*B*;supplementary fig. 7, Supplementary Material online); For *PH02Gene38072*, it transformed through multiple steps including four substitution and one indel mutation (fig. 3*C*;supplementary fig. 8, Supplementary Material online). Totally, there were seven de novo genes formed by substitution like that and the remaining 12 genes by indel mutation in a similar way (supplementary table 9, Supplementary Material online). Furthermore, all the de novo genes had transcriptional evidence supported by full-length transcriptome data in *P. edulis* and/or *Bo. amplexicaulis*. Among them, 11 genes also had transcriptional evidence in *Ol. latifolia*, suggesting that the noncoding RNA transcription had emerged earlier than the open reading frames (ORFs) in evolution, similar to *Oryza* (Zhang et al. 2019).

We further examined the 19 de novo genes for their potential functionality and particularly from the translational evidence. Firstly, we calculated the substitutions at synonymous sites (Ks), nonsynonymous sites (Ka) and Ka/Ks value of orthologs between *P. edulis and Bo. amplexicaulis*. Among them, we detected nine genes with Ka/Ks value significantly less than one and two genes significantly larger than 1 (*P* < 0.05, *χ*^2^ test) (supplementary table 10, Supplementary Material online), indicating purifying and positive selection, respectively. These results suggest they are undergoing evolutionary constraints and thus support the coding potential of these de novo genes. We subsequently analyzed the expression patterns of all de novo genes in the seven *P. edulis* tissues and found that all of them were tissue-specific expressed (fig. 3*D*) like the expression pattern of de novo genes in rice (Zhang et al. 2019). The tissue with the largest number of specifically expressed genes (SEGs) was the shoot with 13 genes and the second one was the inflorescence with four genes. Moreover, the shoot-specific genes *PH02Gene15427*, *PH02Gene17673*, *PH02Gene28800*, and *PH02Gene36478* were highly expressed with FPKM > 1,000 at the stage of 1.0−7.0 m and had the mass spectrometry (MS) proteomics peptides (supplementary tables 11 and 12, Supplementary Material online). *PH02Gene36478* (fig. 3*B*) is an example with six peptides, supported by liquid chromatography-tandem MS (LC−MS/MS) in the shoots (supplementary fig. 9, Supplementary Material online). These results lend further support for that the new genes particularly de novo genes could be related to the fast growth of woody bamboo shoots.

**Fig. 4.**
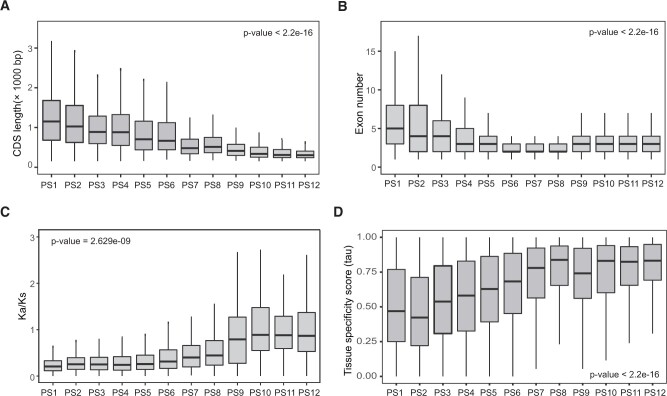
Patterns of new genes in evolution, expression and gene structures. (*A*) Younger genes are shorter than older genes. (*B*) Younger genes have fewer exons than older genes. (*C*) Evolutionary rates of young genes are higher than old genes. (*D*) Younger genes are more tissue specific expression. A Kruskal−Wallis rank sum test was used to determine significance.

In general, we found that these de novo genes and orphan genes experienced gradual evolutionary processes with their ages in terms of their exon−intron structure, protein length, and expression tissue specificity. The features of gene length, exon number, evolutionary constraint, and tissue-specific expression all showed clear age-dependent trends (*P* < 0.01 in the Kruskal−Wallis rank sum test) (fig. 4; supplementary table 13, Supplementary Material online). In contrast to old genes, new genes tended to possess shorter CDS sequences (a median value of 303 bp for PS12 vs. 1,179 bp for PS1) (fig. 4*A*) and less exons (three exons of PS12 vs. five ones of PS1) (fig. 4*B*) and evolved with less evolutionary constraint (Ka/Ks) (0.92 vs. 0.26) (fig. 4*C*). The new genes also showed higher expression specificity (0.83 vs. 0.47) (fig. 4*D*). These observations reveal well the stepwise evolution of novel gene structures: New genes gradually recruited more exons, expanded their exon lengths, enhanced evolutionary constraint, and acquired the ability of more broad expression.

### Divergent Expression of WGD Duplicated Genes in Shoot

**Fig. 5.**
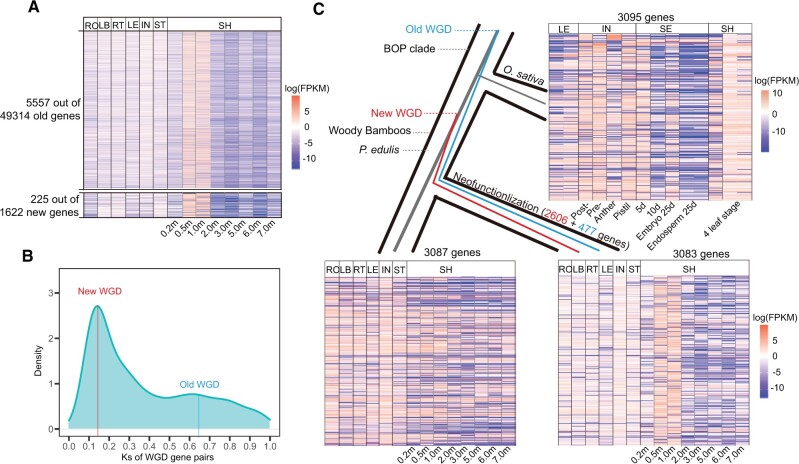
Shoot SEGs and their evolutionary model. (*A*) SEGs of shoots in *P. edulis*. RO, root; LB, leaf; RT, rhizome tip; LB, lateral bud; IN, inflorescence; ST, shoot tip; SH, shoot. (*B*) Ks distribution of shoot biased related WGD-pairs in *P. edulis*. (*C*) Evolution of expression pattern of SBW. SBW, shoot biased WGDs.

As the observation of shoot-specific expression of de novo genes, we further examined the SEGs at the whole genome level across the *P*. *edulis* tissues, which may provide clues about tissue-specific and gene-specific biological functions (Favery et al. 2001; Wagner and Lynch 2010; Borg et al. 2011). Totally, we identified 7,013 SEGs, including 574 in roots, 43 in later buds, 356 in leaves, 22 in rhizome tip, 194 in inflorescence, 42 in shoot tips, and 5,782 in shoots (table 1; supplementary table 14, Supplementary Material online). A majority of 82.45% of detected SEGs was found in shoots and concentrated in the stage of 0.5–1.0 m height with 4,227 out of 5,782 shoot-SEGs (fig. 5*A*). The large number of SEGs identified for shoots might be caused by the more samplings of this tissue with eight developmental stages than those of the other six tissues with each just having one stage. To assess this possibility, we performed eight simulations for identifying SEGs with each one only selecting one developmental stage of shoots (supplementary table 15, Supplementary Material online). Similar results were obtained when selecting the samples of shoots at 0.5 m (65.61% of the total 6,305 SEGs identified in shoot) and at 1.0 m (64.43% of 6,121 SEGs) height whereas there were still higher proportion of SEGs (20.35−36.23%) identified in shoots for the remaining stages despite not significantly for all of them. Therefore, we can conclude that the shoots indeed have more tissue-specific expressed genes, not only for the 19 de novo genes but also for all the genes in the genome, especially at the stages of 0.5 and 1.0 m. The shoot SEGs were enriched in functions involving the plant-type cell wall organization, biogenesis and mitochondrial mRNA processing (supplementary fig. 10, Supplementary Material online).

**Table 1.** The Number of SEGs in Seven *Phyllostachys edulis* Tissues.

PS	Root	Lateral bud	Leaf	Inflore-scence	Shoot tip	Rhizome tip	Shoot	Total
PS1	163	10	143	65	10	3	1,389	1,783
PS2	135	11	88	48	15	3	1,793	2,093
PS3	81	9	71	23	3	4	609	800
PS4	120	10	47	45	8	7	1,049	1,286
PS5	16	2	0	0	1	1	138	158
PS6	36	1	0	0	1	4	389	431
PS7	6	0	0	0	0	0	43	49
PS8	7	0	0	0	1	0	123	131
PS9	2	0	1	0	0	0	24	27
PS10	2	0	1	2	0	0	35	40
PS11	2	0	1	3	2	0	36	44
PS12	4	0	4	8	1	0	154	171
Total	574	43	356	194	42	22	5,782	7,013

We also identified SEGs for rice in four tissues (supplementary table 16, Supplementary Material online) for comparison and found that the tissue having the most SEGs was the inflorescence (54.45% of a total 3,242 SEGs) (supplementary table 17, Supplementary Material online). This was common for reproductive organs to have more tissue-specific genes as found in many species of animals and plants (Wu et al. 2014; Gossmann et al. 2016; Zhang et al. 2018; Fang et al. 2020), in sharply contrast to the situation found in the woody bamboos. Moreover, the identified 5,782 shoot-SEGs above included 5,557 old genes out of a total 49,314 (PS1−PS9) and 225 new genes out of a total 1,622 (PS10−PS12). The possibility of being specifically expressed in the shoots was slightly higher for new genes than for old genes (14.06% vs. 11.34%, *χ*^2^ = 10.318, *P *=* *0.0013).

In considering the WGD history of woody bamboos (Peng, Lu, et al. 2013; Guo et al. 2019), we implemented the Dup_GenFinder pipeline (Qiao et al. 2019) to infer the origin of shoot-SEGs. We found that 3,083 genes (53.32% out of 5,782 shoot SEGs) were derived from the WGD events with paralogs in the genome, and 28.09% of them had all copies whereas the remaining had only one copy specifically expressed. The WGD genes were significantly enriched in the shoot-SEGs (*P* < 2.2e−16 of Fisher’s exact test). Among them, 2,606 genes could be attributed to the recent independent WGD event of woody bamboos at 22 Ma (Peng, Lu, et al. 2013; Guo et al. 2019) and the remaining 477 ones to the old ρ WGD event shared by the grass family (fig. 5*B*) (Ma et al. 2021). We further investigated the evolution of gene expression for these WGD-duplicated genes by assuming that the expression patterns in the rice without recent WGD represented the ancestral state. Based on the collinearity between the genomes of *P. edulis* and rice, 3,095 orthologous genes were identified in the rice, which corresponded to shoot-biased WGDs. There were only 457 tissue-specific genes whereas 2,638 genes were broadly expressed across the different tissues (fig. 5*C*). Moreover, 322 of these 457 tissue-specific genes came from the inflorescence tissues as expected. On the other hand, nearly all of the 3,087 paralogous genes to the shoot-SEGs within *P. edulis* were also broadly expressed with only 49 of them being tissue-specific. For the 3,083 shoot-biased expression WGD duplicates, we only detected 10 shoot-SEGs with Ka/Ks value more than 1 ((Ka/Ks ≥ 1) = 10/3,083 = 0.0032), suggesting that obtaining a duplicate pseudogene is an event with a significantly small probability (supplementary fig. 11 and table 18, Supplementary Material online). This suggests that most gene pairs were underlying purifying selection, therefore their translated products are not functionless. Together, these results suggested that the divergence of gene expressions for the WGD duplicated genes in woody bamboos were accompanied by one of duplicated copies being specifically expressed in the shoot.

### Evolution of New Genes by Coexpression with Old Genes

**Fig. 6.**
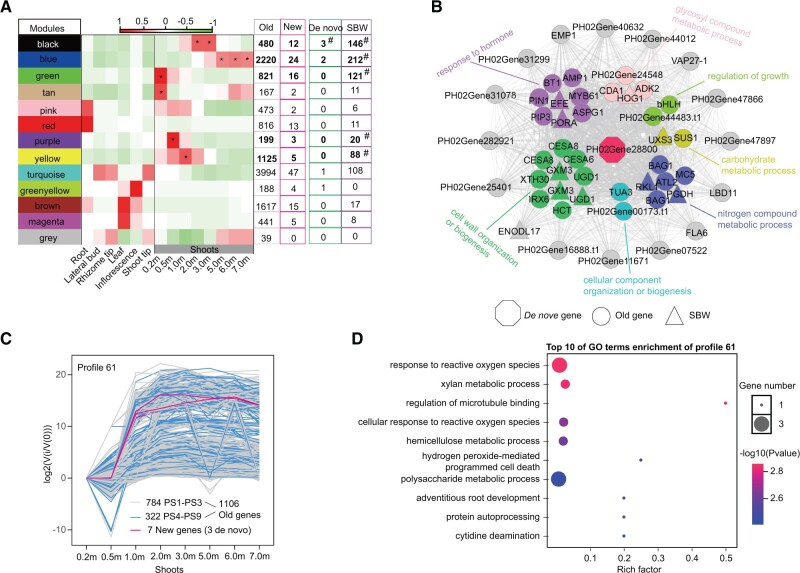
Coexpression of new genes and old genes. (*A*) Gene coexpression network identified by WGCNA. Thirteen coexpression modules were detected, including black, blue, green, tan, pink, red, purple, yellow, turquoise, greenyellow, brown, magenta, and gray modules. The “*” represent modules correlated with shoots when the correlation value is more than 0.50 and *P* value <0.01. The ^“#”^ represent de novo genes and SBW genes significant enrichment in module when the *P* value of Fisher’s exact test < 0.01. (*B*) A de novo gene *PH02Gene28800* centric coexpression networks*.* The top 50 correlated genes were shown in the network. Colors refer to GO enrichment terms. Gray refers to genes without enriched in any GO terms. The shapes refer to gene compositions. (*C*) Profile 61 showing high overexpression at 1.0–3.0 m stages of shoot by STEM analysis. Colors refer to gene ages. (*D*) The GO enrichment of the Profile 61.

To acquire their new functional roles, new genes need to be integrated into the gene interaction network, which can be inferred from gene expression data (Zhang et al. 2015). Using the weighted gene coexpression network analysis for the samples of shoots at eight different developmental stages and the other six tissues (Langfelder and Horvath 2008), we estimated correlations between genes across transcriptome samples and clusters genes with similar profiles into modules. We clustered 12,728 genes into 13 modules (black, blue, green, tan, pink, red, purple, yellow, turquoise, greenyellow, brown, magenta, and gray modules) ranging in size from 39 to 4,041 genes (fig. 6*A*;supplementary table 19 and fig. 12, Supplementary Material online). Among these modules, six of which (black, blue, green, tan, purple, and yellow modules) were found to be related to the shoots (supplementary figs 13 and 14, Supplementary Material online). These shoot-specific networks included from 169 to 2,244 genes and were generally enriched for genes belonging to different synthetic and catabolic pathways, including RNA biosynthetic and metabolic process and response to stimulus at 0.2 m, chromatin organization at 0.5 m, transport and translational elongation at 1.0 m, cell wall organization at 2.0 and 3.0 m, and biogenesis from 5.0 to 7.0 m (supplementary fig. 15, Supplementary Material online).

All the 13 modules except gray module harbored both old genes and new genes (fig. 6*A*), whereas the five modules (black, blue, green, purple, and yellow) with enrichment of WGD-duplicated genes with divergent expression patterns were all shoot-specific (*P* value of the Fisher’s Exact Test < 0.01). Even the black module at the stages of 2.0 and 3.0 m in height was meanwhile enriched for de novo genes (*P* < 0.01 in Fisher’s Exact Test). These results suggested that new genes were coexpressed with a large number of old genes in the shoot, presumably recruited into the networks through WGD-duplicated genes showing shift of expression toward shoots. Taking the module enriched for de novo genes as an example for more details, we found that *PH02Gene28800* was one of the three de novo genes in the black module originating in the common ancestor of woody bamboos. According to the weighted values connected to this gene, we selected 50 genes to display the network (fig. 6*B*). Among these genes, ten genes were WGD-duplicated genes specifically expressed in the shoot and the remaining 40 ones were evolutionarily conserved old genes. The functional enrichments of these genes were cell wall organization or biogenesis (ten genes), response to hormone (eight genes), glycosyl compound metabolic process (four genes), and regulation of growth (two genes) (supplementary table 20, Supplementary Material online). These pathways were all previously reported to be involved in the fast growth of woody bamboo shoots (Paque et al. 2014; Li et al. 2018), underlying how the new genes to function in the fast growth of bamboo shoot by interacting with the existed genes.

To further check whether the eight different developmental stages of shoots have similar trends of coexpression for new and old genes, we clustered expression profiles of shoots by Short Time Series Expression Miner, which was designed to analyze time series with three to eight points (Ernst and Bar-Joseph 2006). In total, 13,001 genes were clustered in to 19 expression patterns (*P* < 0.05), and 12 of which were comprised of both old and new ones (supplementary fig. 16, Supplementary Material online), showing a general similar coexpression pattern of new genes and old genes among different stages of shoots. Among them, Profile 61 harbored 7 new genes and 1,106 old genes (784 from PS1−PS3 and 322 from PS4−PS9) with overexpression at the 2.0 and 3.0 m stages (fig. 6*C*). Within the seven new genes of Profile 61, there were three de novo genes (*PH02Gene15427*, *PH02Gene17673*, and *PH02Gene28800*). GO function enrichments of Profile 61 were mainly involving the xylan metabolic process and hemicellulose metabolic process, both of which were cell wall related (fig. 6*D*). The cell wall metabolism was involved in the fast growth of woody bamboo shoots (Cui et al. 2012; He et al. 2013; Peng, Zhang, et al. 2013; Wang et al. 2019), further suggesting the important roles of the evolution of coexpression for new genes and old genes in the emergence of this unique trait of woody bamboos.

## Discussion

Evolutionary new genes have been seen as one of major drivers in phenotypic evolution (Khalturin et al. 2009; Kaessmann 2010; Tautz and Domazet-Loso 2011; Chen et al. 2013). The fast growth of woody shoot and consequent tall stems in bamboos, a key innovation and distinguishing trait within the grass family (Song et al. 2011; Lima et al. 2012; Buckingham et al. 2014), provides an opportunity to explore the role of new genes in the morphological diversification of the grass species. A novel cell type called long parenchyma cells was even evolved in the woody bamboo shoot (He et al. 2002; Gritsch and Murphy 2005; Wei et al. 2018). With definite phenotypic innovations and recent WGD events (Guo et al. 2019), which provide major sources of new genes (McLysaght et al. 2002; Long et al. 2003; Kellis et al. 2004; Edger et al. 2015; Clark and Donoghue 2018), the bamboos can be an ideal system to study the connection between genomic novelty and evolutionary innovation. Nevertheless, to date, a comprehensive investigation of the genomic basis underlying the evolution of fast growth of woody shoot in bamboos is still lacking, with existing studies mostly focusing on a few genes and their associated gene families (Peng, Lu, et al. 2013; Peng, Zhang, et al. 2013; Wei et al. 2018, 2019). By collecting genomic and transcriptome data of bamboos, our phylostratigraphic and transcriptomic analyses here have enabled us to acquire a systematic view of bamboo shoot evolution at the genome level.

With sequenced genomes representing the four major lineages of Bambusoideae (Guo et al. 2019), a total of 1,622 orphan genes were identified in bamboos by using phylostratigraphy, with the highest birth rate for new genes coinciding with the origin of this subfamily. By linking gene ages to expression, both higher TAI and TDI values suggest that the transcriptomes of shoots are young and divergent in evolution among the various bamboo tissues. These include vegetative organs such as leaf and root, as well as reproductive tissue of inflorescence. Moreover, the new genes originating in the common ancestor of woody bamboos (PS11) show the highest expression level among genes of different phylostrata and in the shoot at the developmental stage of 2.0 m. This is in sharp contrast to that the new genes are generally lower expressed than old genes (Donoghue et al. 2011; Palmieri et al. 2014; Zhao et al. 2014), which contains many housekeeping genes, although the expression level of old genes is higher than that of new genes in other tissues of bamboos. In addition to young transcriptome, a large number of genes are specifically and highly expressed in the shoots, particularly at the stages of 0.5 and 1.0 m. The majority of these shoot biased expressed genes are derived from the duplicated genes of WGD with one copy experiencing the divergent expression patterns from the ancestral state in the shoot.

Furthermore, within the origin mechanisms of new genes the de novo origin is probably the most exciting and these genes may play a key role in the evolution of innovative trait (Van Oss and Carvunis 2019; Blevins et al. 2021). To avoid potential sources of orphan genes alternative to de novo origination in the phylostratigraphic analysis (Moyers and Zhang 2016; Domazet-Loso et al. 2017; Vakirlis et al. 2020), we used a strict procedure of de novo gene identification that requires reconstruction of noncoding ancestors and processes of de novo origination (Zhang et al. 2019). We identified 19 candidate de novo genes in the woody bamboos with significantly similar ancestral noncoding sequences and reconstructed the origination processes of these de novo genes. As expected, these genes are all specifically expressed in individual tissues as suggested for de novo genes previously (Levine et al. 2006; Toll-Riera et al. 2009; Schlotterer 2015). Interestingly, most of them and 13 genes were found to be expressed in the shoots at developmental stages from 0.5 to 2.0 m and the following tissue was the inflorescence but with only four de novo genes expressed. Altogether, we can conclude that the evolution of woody bamboo shoot closely correlated with genomic novelties from highly expressed orphan genes (including de novo genes) to altered expression patterns for WGD duplicated genes. Moreover, these events mainly occur in the stages of 0.5−2.0 m, a key transition point for *P*. *edulis* from slow to fast growth (Xu et al. 2011; Song et al. 2016). As such, the development of woody bamboo shoot shows an “inverse hourglass” model for the evolutionary age of the transcriptome with old genes expressed at early and late stages. This is in contrast to the common hourglass model as found firstly for embryogenesis (Domazet-Loso and Tautz 2010; Quint et al. 2012; Levin et al. 2016), and afterwards many postembryonic phases of plant development, with a phylotypic stage expressing the oldest transcriptome set (Drost et al. 2016; Leiboff and Hake 2019; Trible and Kronauer 2021). The transition stage from slow to fast growth may reflect the most unique features of shoot and thus the morphological and molecular patterns are coupled in the woody bamboos.

The observation of shoot serving as a major source of genomic novelties in woody bamboos is intriguing. A theory of “out-of-testis” for the emergence of new genes was firstly proposed in animals (Levine et al. 2006; Vinckenbosch et al. 2006; Kaessmann 2010). Biased expression of new genes in male reproductive tissues has also been identified afterwards in the model plants of *Arabidopsis* and rice (Cui et al. 2015), as well as recently in nematodes (Rodelsperger et al. 2021). Several hypotheses have been put forward to explain this phenomenon and may be driven by a common evolutionary force of male gametophyte competition (Cui et al. 2015; Wang et al. 2016). However, in the woody bamboos, the shoot rather than the reproductive tissues acts as an “innovation incubator” for the evolution of new genes. A potential explanation is that, as polyploid plants, the woody bamboos often reproduce vegetatively with the complex system of rhizomes and shoots playing an important role in propagation, and use sexual reproduction occasionally with long flowering cycles of 20−60 years in general (Janzen 1976).

With an enrichment of genomic novelties in the growth of woody bamboo shoot revealed, these novelties represent a mix of products of new genes as well as WGD duplicated genes undergoing divergent expression. Gene duplication through WGD is a major source for generating new genes and function (Conant and Wolfe 2008; Innan and Kondrashov 2010; Zhen et al. 2012; Sandve et al. 2018), but other process such as de novo formation can also generate new genes (Zhang et al. 2019; Blevins et al. 2021). And our identified new genes here are all resulted from other processes rather than WGD duplication. In particular, there are 19 genes of de novo origin, the first case described in bamboos, and the majority of them are related to the evolution of woody bamboo shoot. On the other hand, the WGD duplicated genes are also involved in this innovation of woody bamboos with rapid divergence of expression in the shoot for one copy, pointing to potential functional novelty (Taylor and Raes 2004; Braasch et al. 2016; Sandve et al. 2018). In all, the combination of new genes and WGD duplicated genes forms the molecular basis of the innovation of fast woody shoot growth in bamboos. This extends our understanding of novel gene and function formation in bamboos and will provide an important resource for future studies of gene function.

Furthermore, we demonstrated the gene interaction between new genes and old genes by the WGCNA analysis, and many of the identified coexpression modules are enriched in genes involving the process of cell wall metabolism, cell cycle regulation, and plant hormones network. All of these processes are closely related to the rapid growth of woody bamboo shoot (Cui et al. 2012; He et al. 2013; Peng, Zhang, et al. 2013; Wang et al. 2019). These results further validate the role of new genes through recruitment into the existing genetic networks in the evolution of woody bamboo shoot. This is somewhat expected as previous studies have shown that new genes should be integrated into and reshape ancestral genetic interaction network to acquire their corresponding biological functions (Zu et al. 2019). In future, more detailed analysis and functional studies of identified new genes and their interacted genes are needed to comprehensively characterize the roles of genomic novelties in the woody bamboo shoots.

## Materials and Methods

### RNA Extraction and Full-Length RNA Sequencing

The samples (leaves and shoots) of *Bo. amplexicaulis and Ol. latifolia* were collected from the same plant for genome sequencing in Guo et al. (2019). Two total RNA samples (leaves and shoots) were extracted using the miRcute Plant miRNA Isolation Kit (DP504) (TIANGEN BIOTECH Corporation, Beijing, China). Total RNA samples were treated with Dnase I to remove DNA contaminant. RNA quantity was determined using Nanodrop, gel electrophoresis, and further by the Agilent 2100 Bioanalyzer (Agilent, Santa Clara, CA). The first-strand cDNA was synthesized using the Clontech SMARTer PCR cDNA synthesis Kit (Clontech). The library was prepared according to the Isoform Sequencing (Iso-Seq) protocol, as described by Pacific Biosciences (PacBio). A total of two single molecular real-time (SMRT) cells were sequenced on the PacBio Sequel platform (Biomarker Tech, Beijing, China). The cDNA library (1−6 kb) was constructed and sequenced using the PacBio Sequel System. Two SMRT cells generated 46.9 Gb raw data.

According to the standard protocol of ISO-seq (SMRT Analysis 5.1.0), raw Polymerase reads that have full passes ≥1 and the predicted consensus accuracy >0.9 were selected (minPasses = 1) (https://github.com/PacificBiosciences/pbbioconda, last accessed October 1, 2020). Then, 941,750 circular consensus sequences (CCSs) were classified into full-length (FL) and non-FL (NFL) CCS according to whether included 5′/3′ cDNA primers and poly (A) tail at the same time. At last, full-length nonchimeric (FLNC) reads were subjected to isoform-level clustering by iterative isoform-clustering for error correction algorithm and herein similar sequences assigned to a cluster. Each cluster was identified as a uniform isoform. NFL cDNA reads were then applied to polish each cluster to produce high-quality isoforms (accuracy > 99%). The difference of 5′ end was not considered when collapsing redundant transcripts. The FLNC sequences were mapped into the genome by Gmap (–cross-species, –allow-close-indels 0) (Wu and Watanabe 2005). Mapped reads were further collapsed by cDNA_Cupcake package (https://github.com/Magdoll/cDNA_Cupcake/wiki, last accessed June 15, 2021) with >85% alignment coverage and 90% alignment identify. The nonredundant FLNC reads were listed in supplementary table 21, Supplementary Material online and the raw data are available at the NCBI SRA archive (PRJNA764002).

### Phylostratigraphic Analysis of *P. edulis and Bo. amplexicaulis*

Gene age was estimated using the genomic phylostratigraphic approach as described previously (Domazet-Loso et al. 2007). Considering the polyploid nature of woody bamboos (*P.* edulis: 2*n* = 4×; *Bo. amplexicaulis*: 2*n* = 6×) (Zhao et al. 2018; Guo et al. 2019), the paralogous genes of *P.* edulis and *Bo. amplexicaulis* were gathered into clusters using OrthoMCL software (Li et al. 2003) with the parameter of I at 1.5, respectively (supplementary fig. 1, Supplementary Material online). Only the longest protein of each cluster was picked up as its representative. Combined representatives of gene clusters with singleton (without paralogs), we generated nonredundant queries for *P.* edulis and *Bo. amplexicaulis*, respectively. Additionally, we download 69 genomes across the Tree of Life to represent 12 evolutionary levels or phylostrata (PS), starting from the origin of cellular organisms (PS1) and ending at the origin of *P. edulis* (PS12) (supplementary table 1, Supplementary Material online).

To evaluate the impact of full-length transcriptome data on gene age analysis, we set up two reference data sets: 1) 69 genomes (data set 1); 2) 69 genomes combined with full-length isoforms of *Bo. amplexicaulis and Ol. latifolia* (supplementary table 22, Supplementary Material online). The addition of full-length transcriptome data mainly affected the number of young genes. Finally, we compared redundant query sequences of *P. edulis and Bo. amplexicaulis* with other 68 genomes and full-length transcriptome data via BLASTp and TBlastN algorithm with an e-value 10^−4^ threshold, and then assigned all protein-coding genes into 12 phylostrata. The methods and parameters were consistent with previous studies (Domazet-Loso et al. 2007). According to phylostratigraphic method, if a putative homolog of one gene was first identified in one phylostratum, it was assumed as the age of that gene. If no BLAST hit was detected in other phylostata, the corresponding protein was assigned to the youngest PS (PS12). Gene cluster fixed rates were calculated according to the formula, *r* = *N*/*T*: *r* representing the number of clusters per My; *N* representing the number of clusters in the phylostratum; *T* being the duration of the interval My in the phylostratum. Based on the cluster results of OrthoMCL, gene copies of each cluster were classified into one of the following three groups by its family size in the clustering according to Guo (2013): 1) A singleton is a single-copy gene; 2) a two-gene family has two copies; and 3) a multigene family has ≥3 copies.

### Gene Expression Quantification and Proteomic Peptides Identification

We obtained published RNA-seq data for *P. edulis* from NCBI SRA databases (Gao et al. 2014; Zhao et al. 2016; Wang et al. 2017, 2019) (supplementary table 4, Supplementary Material online). Briefly, we trimmed reads using the Trimmomatic (Bolger et al. 2014) after analysis in FASTQC (https://www.bioinformatics.babraham.ac.uk/projects/fastqc/, last accessed July 14, 2021), and then computed expression level for each gene using RSEM v1.2.16 (Li and Dewey 2011) with Bowtie2 aligner (Langmead and Salzberg 2012). Read counts of gene expression data were removed batch effect by the removeBatchEffect function in Limma package (Ritchie et al. 2015).

We also downloaded transcriptome data of *D. sinicus*, another tropical woody bamboo species, under the accession of PRJNA418355 (Chen et al. 2018). Because without reference genome of *D. sinicus*, the clean reads were assembled into contigs using the program Trinity v2.4.0 (Grabherr et al. 2011). The transcript abundance was quantified using software RSEM v1.2.16 software (Li and Dewey 2011) with Bowtie2 aligner (Langmead and Salzberg 2012). The protein of *D. sinicus* transcripts was predicted using TransDecoder (http://transdecoder.sourceforge.net/, last accessed July 30, 2021).

The available MS raw data of leaf and seeding (Yu et al. 2019), and shoot (Tao et al. 2020) were download. MaxQuant 1.6.17.0 (Tyanova et al. 2016) was used to perform a database search against the moso bamboo protein database to identify peptides.

### Transcriptome Index Calculation

TAI and TDI of each tissue/developmental stage were calculated via myTAI package (Drost et al. 2018). The TAI is a measure that reflects the evolutionary age of a transcriptome at a given ontogenetic stage, where higher values correspond to younger transcriptomes (Domazet-Loso and Tautz 2010). TAI value of a sample is defined as the weighted mean of phylostratum rank *ps_i_* of gene *i* by the expression value *e_is_* in the transcriptome of sample *s*,

$$\mathrm{TAIs}=\frac{\sum_{i=1}^{n}{e_{is}.ps}_{i}}{\sum_{i=1}^{n}e_{is}}$$

where *n* is the total number of genes in the analysis. High TAI indicates that the transcriptome is evolutionarily young, whereas low TAI value suggests the transcriptome is evolutionarily ancient.

The TDI is a weighted mean of Ka/Ks ratios of gene i by the expression value e_is_ in the transcriptome of samples (Quint et al. 2012),

$$\mathrm{TDIs}=\frac{\sum_{i=1}^{n}{\left(\frac{{Ka}_{i}}{{Ks}_{i}}\right).e}_{is}}{\sum_{i=1}^{n}e_{is}}$$

High TDI indicates that the transcriptome is more divergent, whereas low TDI value represents a more conserved transcriptome.

Relative expression (RE) of genes for a given phylostratum (ps) and developmental stage (s) was computed using methods described by Domazet-Loso and Tautz (2010):

$${\mathrm{RE}(\mathrm{ps})}_{s}=\frac{\bar{f}\;-\;{\bar{f}}_{\min}}{{\bar{f}}_{\max}-\;{\bar{f}}_{\min}}$$

where $\bar{f}$ is the average partial concentration of RNAs from phylostratum for a given stage and ${\bar{f}}_{\max}$, ${\bar{f}}_{\min}$ are the maximal and minimal average partial concentration from phylostratum across all considered stages, respectively.

### EP of Highly Expressed Genes

In each tissue, we extracted the 1,000 highest expressed gene of each tissue as the highly expression set (supplementary table 8, Supplementary Material online). Gene age composition of the highly expressed set was counted. The expression ratio (Exp) of a given phylostratum (PS) was calculated in each tissue,

$${\mathrm{Exp}(\mathrm{ps})}_{s}=\frac{\sum_{i=1}^{n}f}{n(ps)}*100$$

where f represented the sum of expression value for a given PS, and *n* was the gene number of a given PS. To assess the expression pattern of genes from different ages across different tissues, we calculated the EP of PS in each tissue,

EP(ps)s=Exp(ps)(n1000)

### Detection of Woody Bamboo De Novo ORFs

Following de novo detective pipeline from Zhang et al. (2019), we identified de novo genes from PS11 genes in *P. edulis* which lack similarity protein sequences out of bamboos. We used nucleotide-to-nucleotide BLAT (minIdentity = 80) to align these 237 *P. edulis* PS11 orphan ORFs to other five related genomes, including other woody bamboo *Bo. amplexicaulis* (Guo et al. 2019), and four outgroup species *Ol. latifolia* (Guo et al. 2019), *R. distichophylla* (Li et al. 2021), *Br. distachyon*, and rice. If exact nucleotide matches covered at least 20% of the corresponding *P. edulis* ORFs, one effective hit was accepted. Only *P. edulis* ORFs had no more than one effective hit in each outgroup species were retained for subsequent analyses. Moreover, we further used BLAT to align these orthologous sequences to *P. edulis* ORFs, to retrieve highly similar orthologous sequences. Some *P. edulis* ORFs were identified as woody bamboo de novo ORFs that had orthologous coding sequences in the *P. edulis* and *Bo. amplexicaulis*, and had noncoding sequences in outgroup species. We used PAML (Yang 2007) to detect the signals (ω = Ka/Ks) of natural selection in de novo orthologous gene pairs between *P. edulis and Bo. amplexicaulis* with runmode = −2 for ML pairwise comparison. To further test whether the ω ratio of a model significantly deviated from neutral evolution (ω = 1), we incorporated the neutral model, which estimates model parameters by fixing ω = 1 (Yang 1997). The statistical significances between the estimated ω and ω = 1 models were estimated by calculating twice the log-likelihood difference following a *χ*^2^ distribution.

To better understand whether the genes across different phylostrata possess various generic features, we analyzed four important genic characteristics. Using custom Perl scripts, we calculated protein length, intron number, and GC content at the CDS level. The Ka/Ks ratio for *P. edulis* versus *Bo. amplexicaulis* was calculated using ParaAT v.2.0 (Zhang et al. 2012) and KAKS_Calculator v.2.0 (Zhang et al. 2006). We calculated the tissue specificity score (tau) of a gene (Yanai et al. 2005; Kryuchkova-Mostacci and Robinson-Rechavi 2017) using the roonysgalbi/tispec package in R package (https://rdrr.io/github/roonysgalbi/tispec, last accessed March 30, 2021).

### Identification Shoot Biased WGDs in *P. edulis*

We used R package “SEGtool” to identify SEGs in each tissue (default parameters, *P* value <0.05) (Zhang et al. 2018). Although SEGtool can detect genes with specifically high and low expression, we only used the SEGs with high expression in this study for further investigation. The number of developmental stages in a certain tissue may affect SEG number. Because the shoot stage contains eight developmental stages, and the other tissues only contain one developmental stage, for fair comparison, we only selected one shoot developmental stage when identifying SEGs for each tissue. We also download rice expression data from http://rice.plantbiology.msu.edu/expression.shtml (last accessed January 15, 2021) to compare SEG patterns with *P. edulis.* Mcscanx (Wang, Tang, et al. 2012) was used to identify syntenic gene pairs between *P. edulis* and rice.

Gene duplications were classified within the *P. edulis* genome using the Dup_GenFinder pipeline (Qiao et al. 2019). This pipeline used the results of an all‐to‐all BLASTp of the *P. edulis* gene set to itself, and BLASTp results comparing the *P. edulis* gene set to a closely related species rice. Dup_GenFinder synthesizes the outputs of these analyses to classify gene duplications into one of five categories, including whole-genome duplication (WGD), tandem duplications, proximal duplications, transposed duplications, and dispersed duplications. Based on the Dup_GenFinder results, the shoot SEGs that match the WGD gene pair were called as shoot biased WGDs (SBW). Then, we calculated the Ka/Ks value for all duplication gene pairs, by using ParaAT v.2.0 (Zhang et al. 2012) and KAKS_Calculator v.2.0 (Zhang et al. 2006).

### Gene Coexpression Analysis by WGCNA and STEM

The R package, WGCNA, was used to perform the weighted correlation network analysis for all 14 ontogenetic stages in R 3.5.3 with the parameters of “softPower = 10, and minModuleSize = 100” (Langfelder and Horvath 2008). Additionally, we extracted the corresponding gene information for each module for further analysis. After the modules were identified, the module eigengene (ME) was summarized by the first principal component of the module expression levels. To construct the developmental stage-specific co-expression network, we regarded sampled tissues and developmental stages as trait. Module–trait relationships were estimated using the correlation between MEs and traits, which allowed efficient identification of the relevant modules. To evaluate the correlation strength, we calculated the module significance (MS), which is defined as the average absolute gene significance (GS) of all the genes involved in the module. The GS is measured as the log10 transformation of the *P* value (log *P*) in the linear regression between gene expression and trait information. In the WGCNA, modules with the highest MS score among all modules are usually defined as the key module and selected for further analysis (Langfelder and Horvath 2008; Wright et al. 2015). Cytoscape v3.6.1 was used to visualize coexpression network (Shannon et al. 2003).

Genes were clustered into co-expression profiles based on their expression dynamics by implementing clustering method STEM v. 1.3.11 (Maximum number of model profiles = 90) (Ernst et al. 2005; Ernst and Bar-Joseph 2006). STEM software uses a nonparametric clustering method to assign genes to predefined expression profiles. It considers expression profiles to be significant if the number of genes assigned to a cluster departs from random. GO enrichment analysis was performed using the clusterProfiler R pakage (version 3.18.1) (Yu et al. 2012).

## Supplementary Material

Supplementary data are available at *Molecular Biology and Evolution* online.

## Supplementary Material

msab288_Supplementary_Data
