# Supplementary material for: New Genes Interacted With Recent Whole-Genome Duplicates in the Fast Stem Growth of Bamboos
Source: Mol Biol Evol. 2021 Sep 28;38(12):5752–68. doi: 10.1093/molbev/msab288 (PMC8662795; doi:10.1093/molbev/msab288)
Supplement: msab288_Supplementary_Data [file msab288_supplementary_data.zip › Supplementary Figures and Tables.pdf]

New Genes Interacted with Recent Whole Genome Duplicates in the Fast Stem Growth of Bamboos

Guihua Jin [1#], Pengfei Ma [1#], Xiaopei Wu[1], Lianfeng Gu[2], Manyuan Long[3*], Chengjun Zhang [1*], De-Zhu Li [1*]

[1] Germplasm Bank of Wild Species, Kunming Institute of Botany, Chinese Academy of Sciences, Kunming, Yunnan 650201, China

[2] Basic Forestry and Proteomics Research Center, College of Forestry, Fujian Agriculture and Forestry University, Fuzhou, Fujian 350002, China

[3] Department of Ecology and Evolution, The University of Chicago, Chicago, Illinois 60637, USA

*** Corresponding authors:** dzl@mail.kib.ac.cn; zhangchengjun@mail.kib.ac.cn; mlong@uchicago.edu

[#] These authors contributed equally.

Supplementary Figures

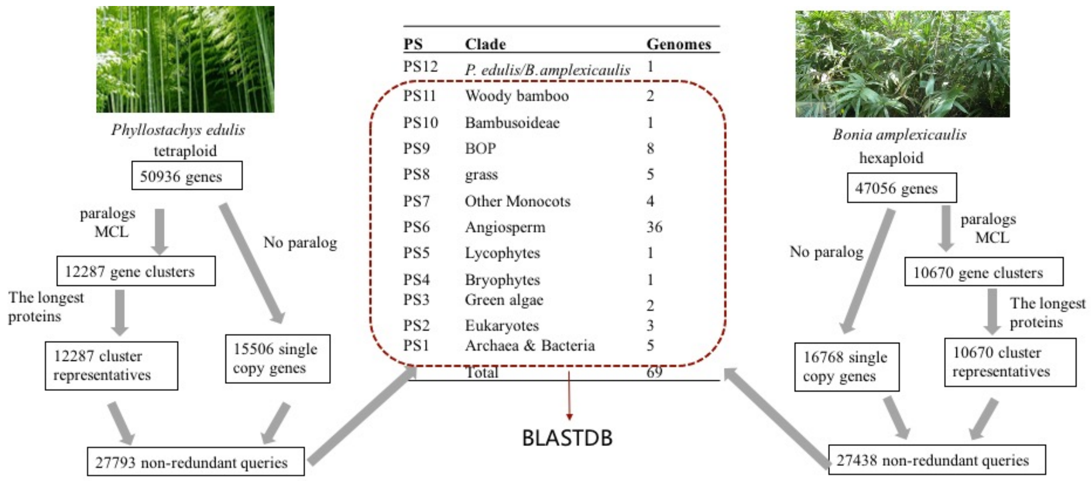

Supplementary Figure 1. Screening process of non-redundant proteins for *Phyllostachy edulis* and *Bonia amplexicaulis*.

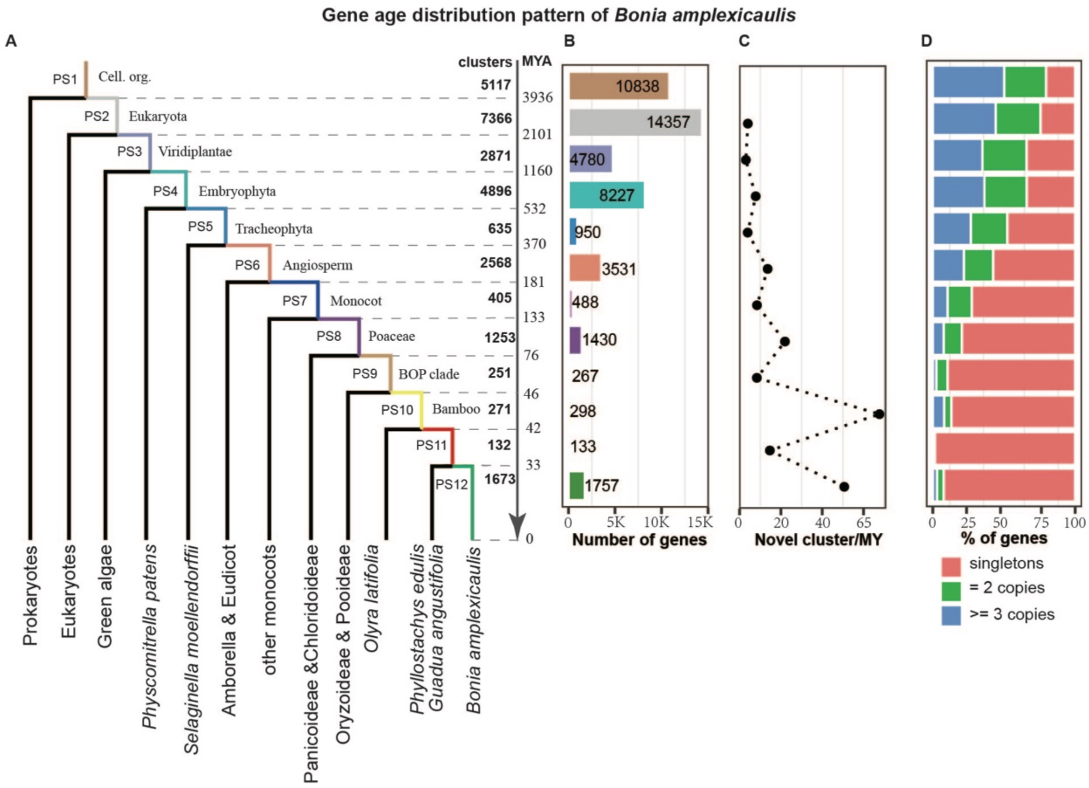

Supplementary Figure 2. Phylostratigraphic age of *Bonia amplexicaulis*. (A) Phylostratigraphic ages distribution. Numbers denote the number of non-redundant gene clusters per phylostratum (PS1-PS12). The tree stratifies species by major evolutionary innovations, from the emergence of simple unicellular organisms up to *P. edulis.* The dating of phylogenetic tree is from TimeTree. Cell. org., cellular organisms. (B) Gene number. (C) Gene fixation rate. (D) Gene copies distribution.

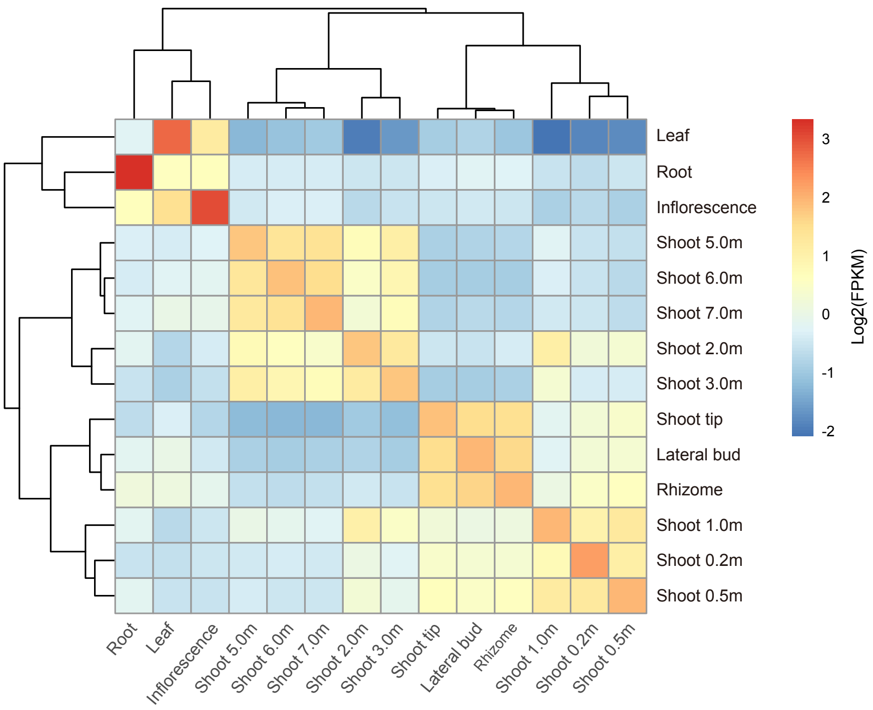

Supplementary Figure 3. The heatmaps show Pearson's correlation coefficients among RNA-seq samples. Each row and column correspond to one RNA-seq sample. The expression levels of all genes were used to calculate the correlation coefficients.

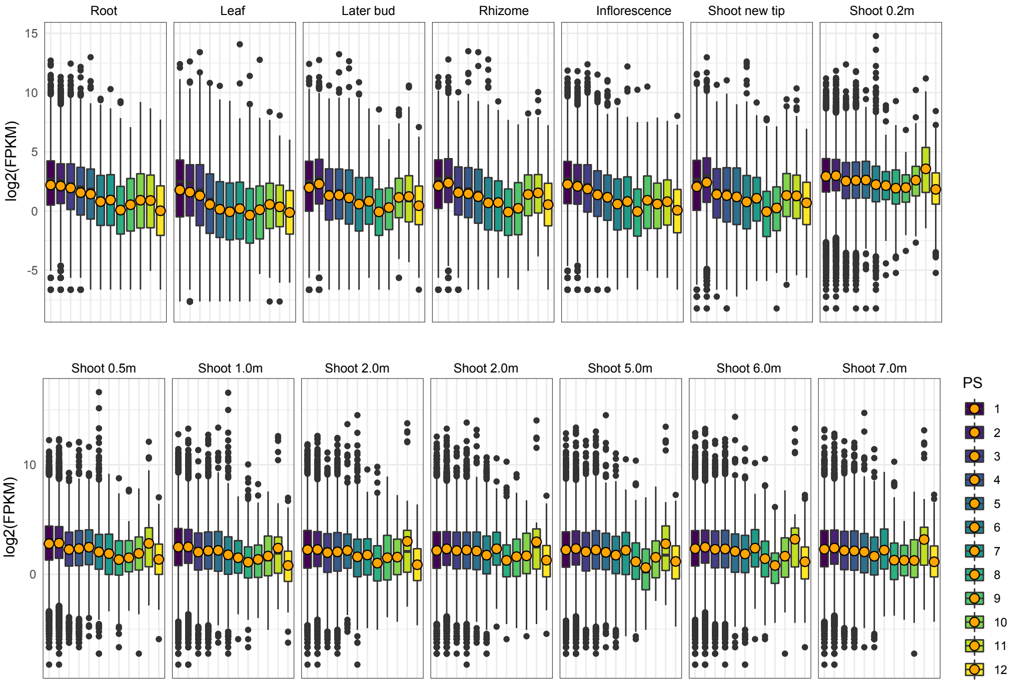

Supplementary Figure 4. Gene expression of each PS across different tissues.

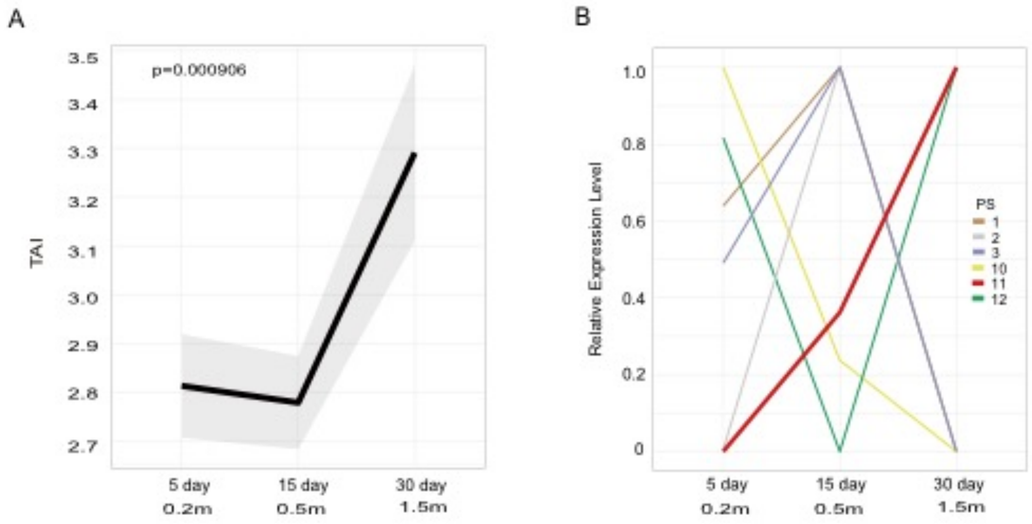

Supplementary Figure 5. TAI profiles of *Dendrocalamus sinicus.* (A)TAI value of across three development stages of *D. sinicus* shoots. (B) Relative expression level for old genes and young genes in *D. sinicus* shoots.

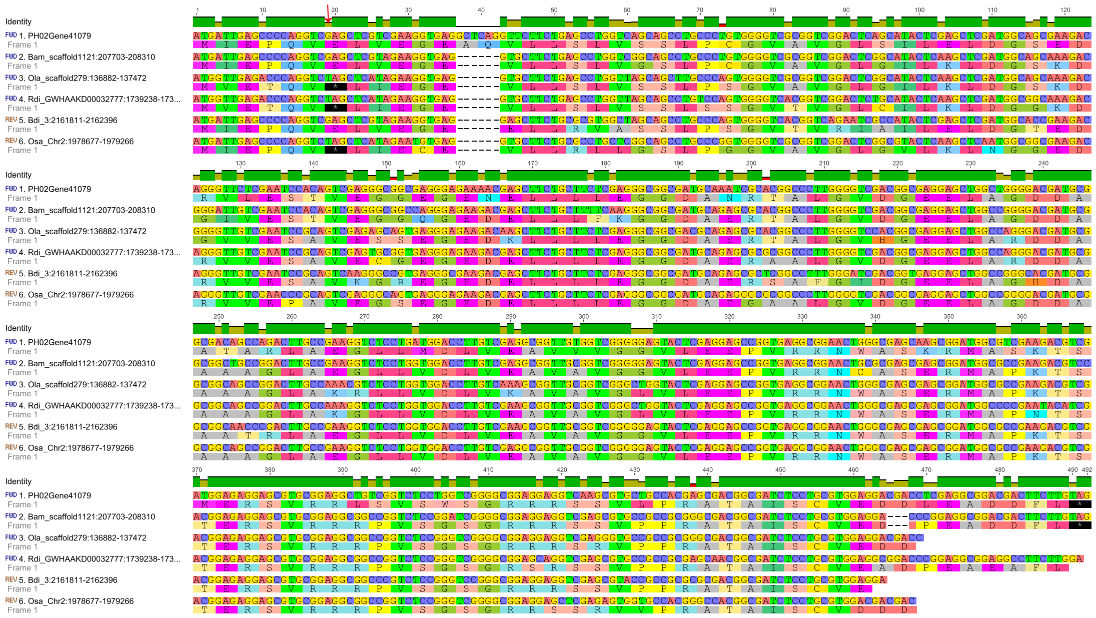

Supplementary Figure 6. The *de novo* formation of the *PH02Gene41079.* In the sequence alignments, the "-" symbol represents added empty positions in the alignment; the black star represents the stop codon; the red arrow represents key substitution to remove premature stop codon of outgroup species.

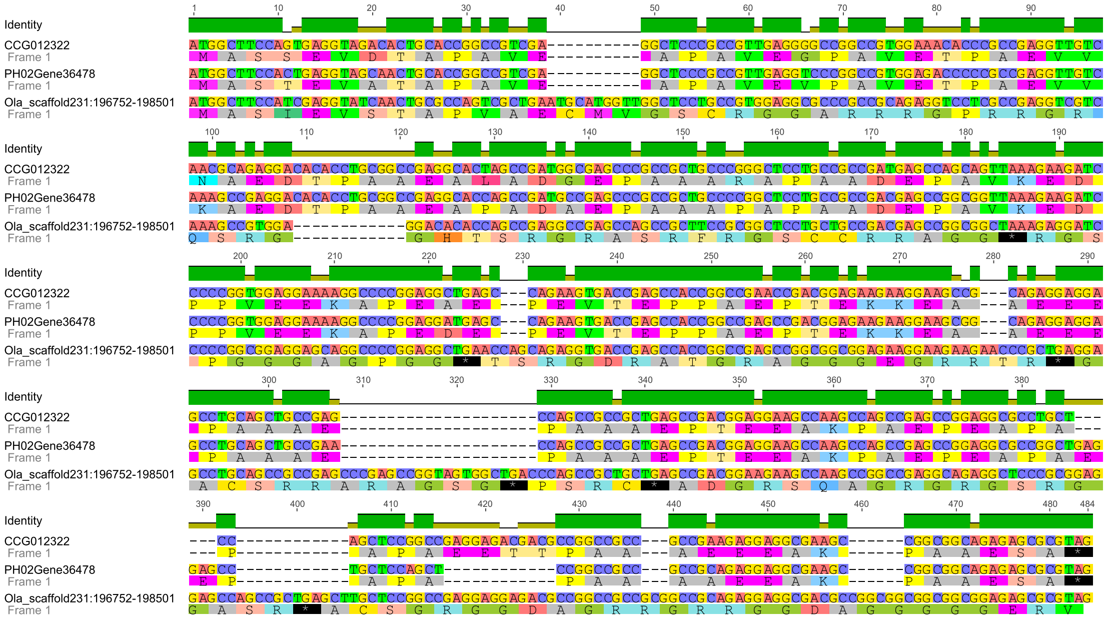

Supplementary Figure 7. The *de novo* formation of the *PH02Gene36478*. In the sequence alignments, the "-" symbol represents added empty positions in the alignment; the black star represents the stop codon; the red arrow represents key substitution to remove premature stop codon of outgroup species.

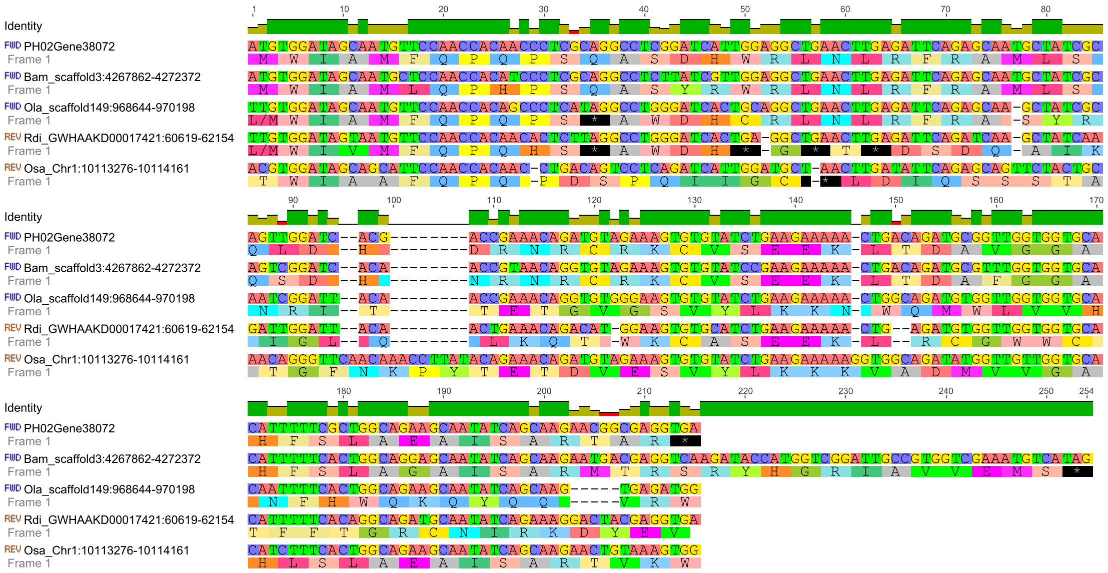

Supplementary Figure 8. The *de novo* formation of the *PH02Gene38072.* In the sequence alignments, the "-" symbol represents added empty positions in the alignment; the black star represents the stop codon; the red arrow represents key substitution to remove premature stop codon of outgroup species.

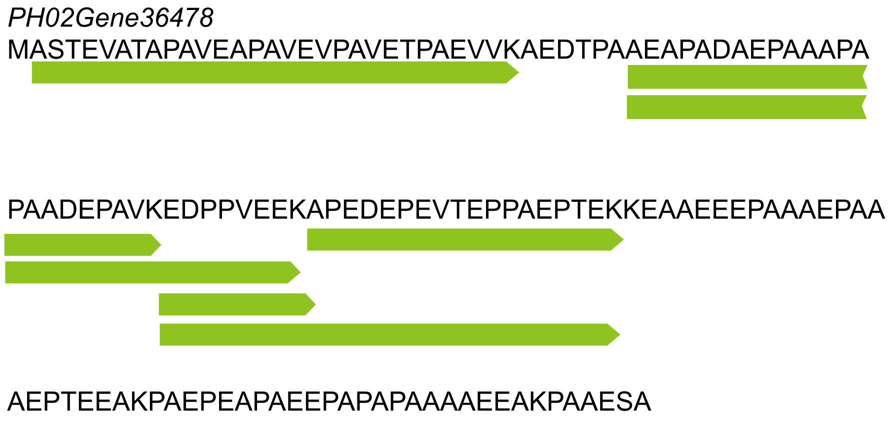

Supplementary Figure 9. Example of *de novo* gene, *PH02Gene36478*, that translate to protein. The peptides (green arrows) detected by Liquid chromatography-tandem mass spectrometry (LC- MS/MS) in the shoots

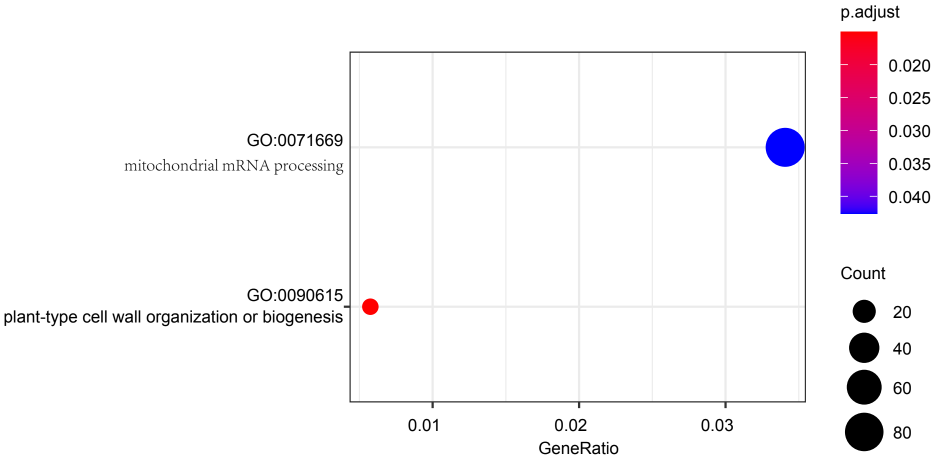

Supplementary Figure 10. GO enrichment of specific expressed genes in shoots.

In this analysis, GO term for biological processes for 5782 specific expressed genes in shoots. The hypergeometric test was used for significance analysis (False discovery rate, FDR <0.05).

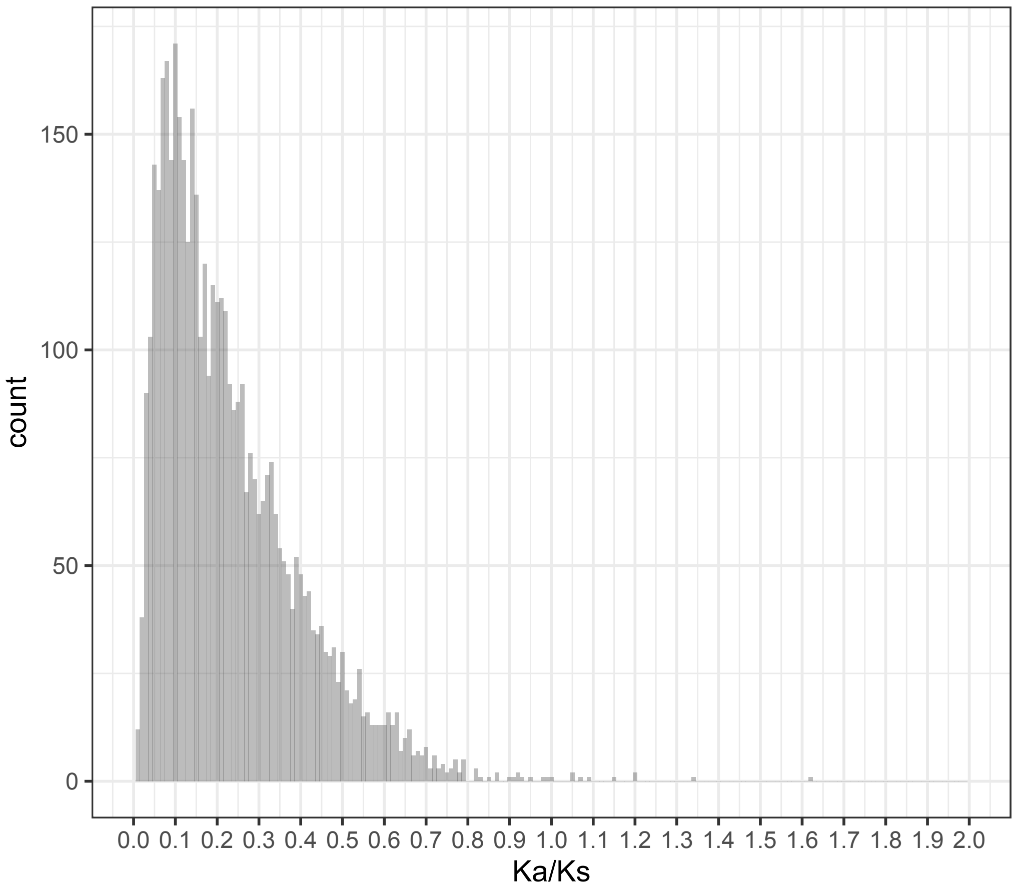

Supplementary Figure 11. The Ka/Ks distribution of shoot biased related WGD-pairs.

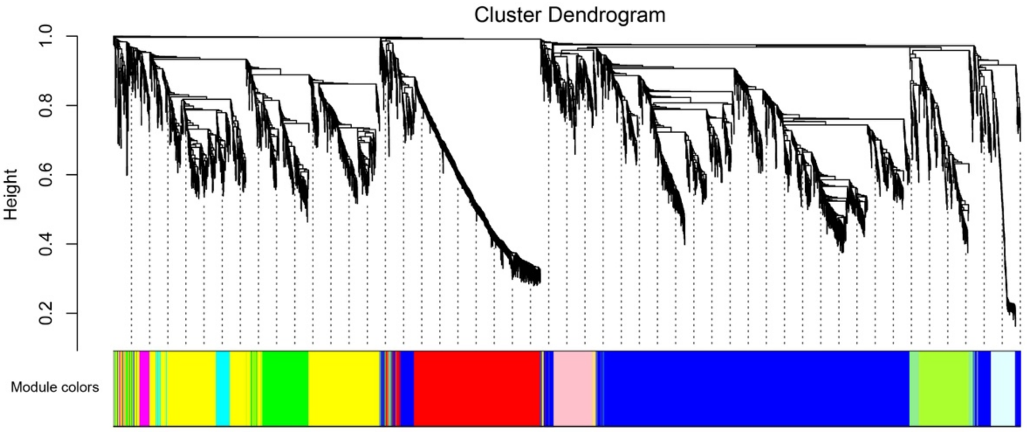

Supplementary Figure 12. WGCNA dendrogram and module colors showing co-expression modules.

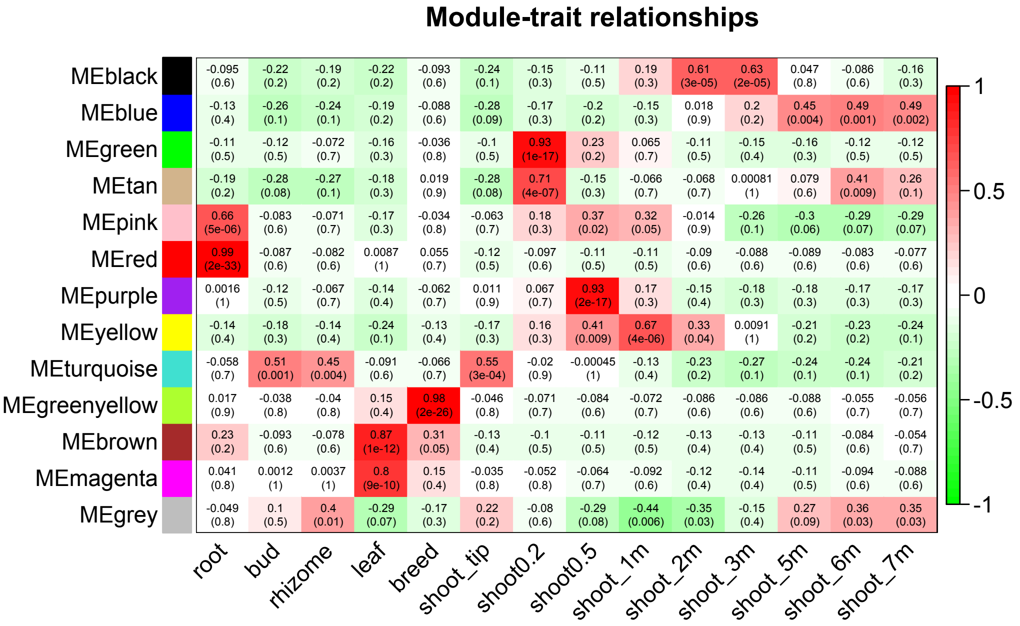

Supplementary Figure 13. Matrix showing Module-Trait Relationships (MTRs) for *P. edulis*. Each row corresponds to a module. Each column corresponds to a tissue or development stage. The MTRs are colored based on their correlation: red indicates a strong positive correlation and green indicates a strong negative correlation

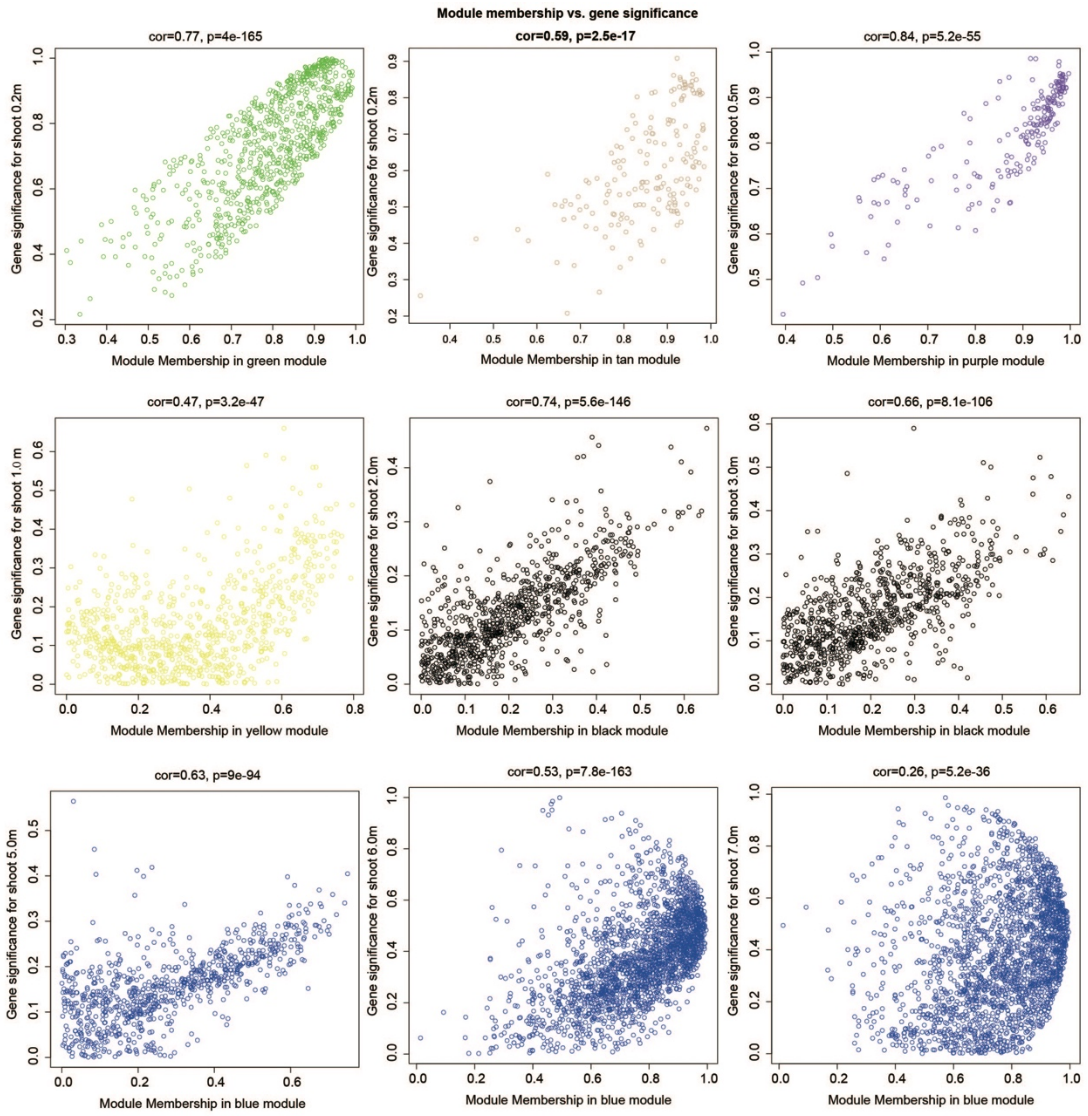

Supplementary Figure 14. Association between the Module Membership and Gene-Trait correlation. Scatterplot of gene significance (*y*-axis) vs. module membership (*x*-axis) in the most significant. In modules related to a trait of interest, genes with high module membership often also have high gene significance.

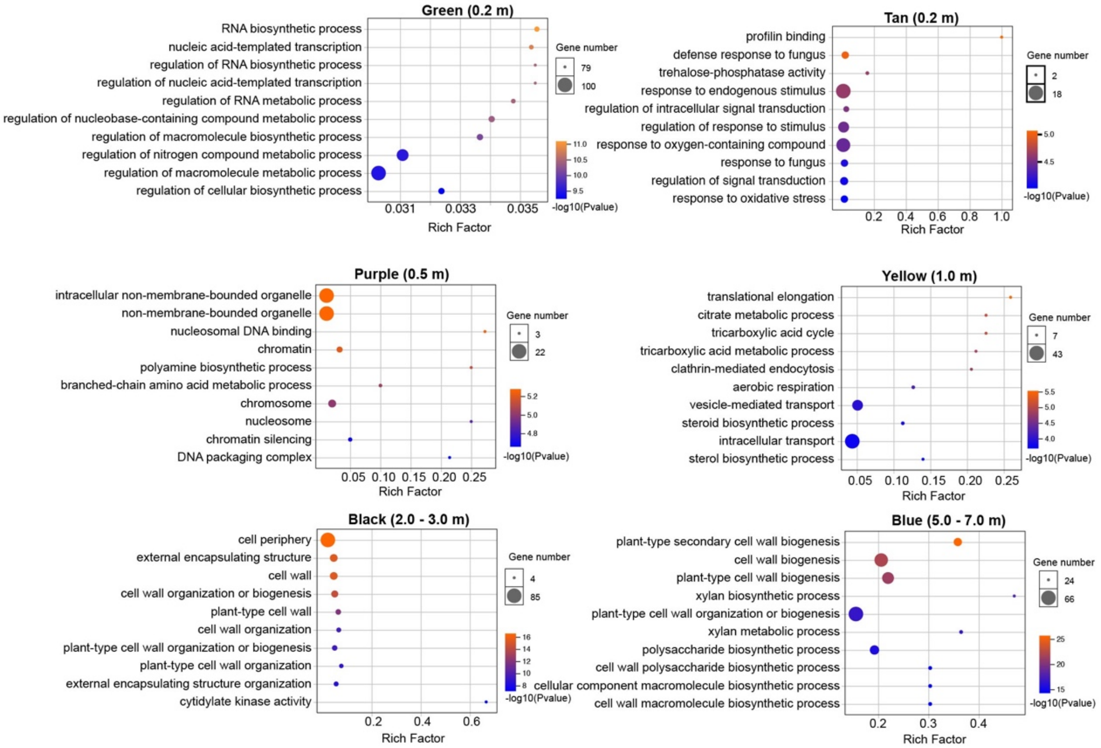

Supplementary Figure 15. The top 10 GO enrichment for shoot-associate modules.
In this analysis, GO term for biological processes for genes in shoot-associated modules. The hypergeometric test was used for significance analysis (False discovery rate, FDR <0.05).

Profile	P89	P0	P61	P1	P36	P33	P60	P85	P82	P88	P84	P16	P63	P75	P6	P29	P52	P19	P4	Total
Model	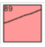	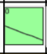	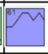	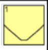	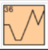	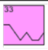	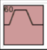	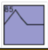	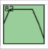	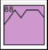	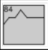	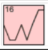	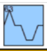	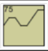	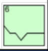	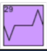	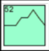	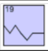	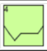	19
ps1	776	872	225	237	196	219	177	221	139	130	113	110	137	127	77	69	51	68	58	4002
ps2	995	752	306	392	269	293	253	217	221	172	159	132	157	110	125	101	86	107	87	4934
ps3	339	160	83	77	85	48	66	31	54	57	38	51	26	43	41	41	16	33	24	1313
ps4	470	296	178	105	105	67	112	62	87	93	57	71	38	48	49	48	19	30	34	1969
ps5	60	33	15	5	10	8	10	11	11	10	3	5	3	3	2	2	1	3	1	196
ps6	100	68	29	16	36	25	25	16	17	26	11	13	8	11	13	17	6	10	7	454
ps7	6	4	5	1	2	1	1	1	1	3	3	3	0	0	0	1	0	0	0	32
ps8	14	6	1	4	4	1	1	1	3	2	3	2	0	1	1	1	0	1	2	48
ps9	1	0	0	0	0	0	0	0	2	0	1	1	0	0	0	0	1	0	1	7
ps10	2	2	0	0	1	0	1	0	2	2	1	0	0	0	1	0	0	0	0	12
ps11	1	1	3	0	0	0	0	0	0	0	1	0	0	0	0	0	0	0	1	7
ps12	4	0	0	0	5	1	0	0	2	5	0	0	0	2	4	0	2	0	2	27
Total	2768	2194	845	837	713	663	646	560	539	500	390	388	369	345	313	280	182	252	217	13001

Legend: >100 | 11-100 | 6-10 | 1-5 | 0

Supplementary Figure 16. Clustered patterns of gene expression across 8 shoot stages identifying by STEM program.

Supplementary Tables

Supplementary **Table 1.** All species used in the blast searches for *Phyllostachys edulis* homologs. All species have been published complete genome sequences.

**PS**	**Phylostatum**	**Genomes**
PS12	*Phyllostachys edulis*	*Phyllostachys edulis*
PS11	Woody bamboos	*Bonia amplexicaulis*, *Guadua angustifolia*
PS10	Bambusoideae	*Olyra latifolia*
PS9	BOP clade	*Triticum aestivum*, *Brachypodium distachyon*, *Hordeum vulgare*, *Aegilops tauschii*, *Triticum dicoccoides*, *Oryza sativa* spp. j*aponica* “Nipponbare”, *Oryza sativa* spp. *indica* “Shuhui 498”, *Leersia perrieri*, (Gramene V60)
PS8	Poaceae	*Sorghum bicolor*, *Zea mays*, *Setaria italica*, *Panicum hallii* 'hal2', (Gramene V60), *Oropetium thomaeum* (phytozome V12)
PS7	Monocots	*Ananas comosus*, *Spirodela polyrhiza*, *Zostera marina*, (phytozome V12), *Musa acuminate* (Gramene V60)
PS6	Angiosperms	*Aquilegia coerulea* v3.1, *Amaranthus hypochondriacus* v1.0, *Arabidopsis halleri* v1.1, *Arabidopsis lyrata* v2.1, *Arabidopsis thaliana* TAIR10, *Boechera stricta* v1.2, *Brassica oleracea capitata* v1.0, *Brassica rapa* FPsc v1.3, *Capsella grandiflora* v1.1, *Capsella rubella* v1.0, *Carica papaya* ASGPBv0.4, *Citrus sinensis* v1.1, *Cucumis sativus* v1.0, *Daucus carota* v2.0, *Eucalyptus grandis* v2.0, *Eutrema salsugineum* v1.0, *Fragaria vesca* v1.1, *Glycine max* Wm82.a2.v1, *Gossypium raimondii* v2.1, *Kalanchoe fedtschenkoi* v1.1, *Kalanchoe laxiflora* v1.1, *Linum usitatissimum* v1.0, *Malus domestica* v1.0, *Manihot esculenta* v6.1, *Medicago truncatula* Mt4.0v1, *Mimulus guttatus* v2.0, *Phaseolus vulgaris* v2.1, *Populus trichocarpa* v3.0, *Prunus persica* v2.1, *Ricinus communis* v0.1, *Salix purpurea* v1.0, *Solanum lycopersicum* iTAG2.4, *Theobroma cacao* v1.1, *Trifolium pratense*, *Vitis vinifera* GeNoscope.12X, *Amborella trichopoda*, (phytozome V12)
PS5	Tracheophyta	*Selaginella moellendorffii* (Gramene V60)
PS4	Embryophyta	*Physcomitrella patens*
PS3	Viridiplantae	*Chlamydomonas reinhardtii*, *Ostreococcus lucimarinus*, (Gramene V60)
PS2	Eukaryota	*Homo sapiens*, *Drosophila obscura*, *Saccharomyces cerevisiae* S288C, (NCBI 20190303)
PS1	Cellular organisms	*Halorubrum* sp., *Haloferax* sp. *MethaNobacterium* sp., *Escherichia coli*, *Staphylococcus aureus*, (NCBI 20190303)

Supplementary Table 3. Gene fixation rates of *P. edulis* and *B. amplexicaulis* gene clusters in each phylostratum.

Species	Phylostratum	Clusters /genes	Duration of the interval (MY)	Average origin rate (clusters/MY)
P. edulis				
	PS1	5857/13485	-	-
	PS2	8117/16632	1835	4.42
	PS3	2982/5180	941	3.17
	PS4	5194/8568	628	8.27
	PS5	603/924	162	3.72
	PS6	2267/3152	189	12.00
	PS7	256/299	48	5.33
	PS8	764/892	57	13.40
	PS9	172/182	30	5.73
	PS10	362/388	4	90.50
	PS11	234/237	9	26.00
	PS12	985/997	33	29.84
Total	12	27793/50936		
B. amplexicaulis				
	PS1	5117/10838	-	-
	PS2	7366/14357	1835	4.01
	PS3	2871/4780	941	3.05
	PS4	4896/8227	628	7.8
	PS5	635/950	162	3.92
	PS6	2568/3531	189	13.59
	PS7	405/488	48	8.44
	PS8	1253/1430	57	21.98
	PS9	251/267	30	8.37
	PS10	271/298	4	67.75
	PS11	132/133	9	14.67
	PS12	1673/1757	33	50.7
Total	12	27438/47056		

Supplementary Table 4. Summary of the RNA-seq data in *Phyllostachys edulis.*

Tissues	SRA Experiments	Total reads	% Mapped
Root	SRR5710697	23196974	94.65
	SRR5710698	21869720	96.38
	SRR5710699	36948986	96.36
Lateral bud	SRR5054408	23491301	94.53
	SRR5054410	22807959	94.65
	SRR5054409	23422633	94.81
Rhizome tip	SRR5054402	23144157	95.92
	SRR5054403	20158804	96.00
	SRR5054404	21542953	96.30
Shoot tip	SRR5054405	21222518	95.28
	SRR5054406	21439077	95.10
	SRR5054407	21598414	92.42
Shoot 0.2 m	SRR6171235	24849920	88.36
	SRR6171236	23269287	88.46
	SRR6171237	13319611	88.04
Shoot 0.5 m	SRR6171238	24116543	88.31
	SRR6171239	21575773	89.45
	SRR6171240	20401496	89.22
Shoot 1.0 m	SRR6171241	25461275	91.93
	SRR6171242	22333404	92.27
	SRR6171243	20765616	92.03
Shoot 2.0 m	SRR6171244	18161807	91.09
	SRR6171245	21410387	91.72
	SRR6171246	19937132	91.43
Shoot 3.0 m	SRR6171247	20689875	90.61
	SRR6171248	19183080	91.13
	SRR6171249	18628730	90.89
Shoot 5.0 m	SRR6171250	17343817	90.88
	SRR6171251	19446688	91.44
	SRR6171252	19681029	91.18
Shoot 6.0 m	SRR6171253	23865483	89.62
	SRR6171254	22614536	90.15
	SRR6171255	21166118	89.86
Shoot 7.0 m	SRR6171256	15684973	90.01
	SRR6171257	19228588	90.55
	SRR6171258	19403361	90.24
Leaf	SRR2035212	40554208	92.40
	SRR2035327	72133675	92.77
	SRR2035263	48689134	92.80
Inflorescence	SRR1187864	67961501	94.33

Supplementary Table 5. Expressed genes and age distribution in *Phyllostachys edulis*.

Tissues	Root	Lateral bud	Leaf	Inflorescence	Rhizome	Shoot tip	Shoot	Total (% all genes)
RNA samples	3	3	3	1	3	3	25	40
**Expressed genes**	**29837**	**26525**	**26281**	**30007**	**27254**	**26084**	**38167**	**41307 (81.10%)**
PS1	8415	7462	7684	8614	7649	7341	10428	11215 **(83.17%)**
PS2	10565	9938	9436	10683	10109	9894	13147	13874 **(83.42%)**
PS3	3172	2593	2598	3086	2719	2556	3853	4250 **(82.05%)**
PS4	4762	3924	3600	4599	4042	3734	6058	6725 **(78.49%)**
PS5	466	373	450	449	404	374	650	712 **(72.06%)**
PS6	1363	1070	1341	1341	1138	1035	2023	2272 **(72.08%)**
PS7	118	106	128	128	111	101	190	220 **(73.58%)**
PS8	301	225	303	303	227	210	501	589 **(66.03%)**
PS9	68	60	68	68	65	64	117	131 **(71.98%)**
PS10	144	171	161	161	182	168	264	279 **(71.91%)**
PS11	105	129	111	131	128	129	189	206 **(86.92%)**
PS12	358	473	401	444	480	477	746	834 **(83.65%)**

The number of expressed genes were determined for each tissue or development stage by an average expression per tissue was > 1 FPKM.

Supplementary Table 7. Gene age composition and expression frequency of the top 1,000 highly expressed genes.

	Root			Lateral bud			Rhizome			Leaf		
PS	Gene%	Exp%	EP	Gene%	Exp%	EP	Gene%	Exp%	EP	Gene%	Exp.%	EP
ps1	38.80	39.61	1.02	37.40	36.28	0.97	36.10	31.86	0.88	38.70	36.26	0.94
ps2	30.40	27.22	0.90	41.30	36.53	0.88	40.00	35.86	0.90	27.20	21.58	0.79
ps3	12.50	12.30	0.98	5.20	3.95	0.76	6.00	4.30	0.72	15.80	21.96	1.39
ps4	11.10	11.35	1.02	9.20	10.29	1.12	10.10	11.17	1.11	9.90	6.64	0.67
ps5	2.00	5.45	2.73	1.50	4.23	2.82	1.70	7.09	4.17	2.80	3.47	1.24
ps6	2.50	2.06	0.82	3.10	5.76	1.86	3.60	6.71	1.86	3.30	2.80	0.85
ps7	0.70	0.45	0.64	0.40	0.28	0.70	0.40	0.44	1.10	0.70	1.43	2.04
ps8	0.70	0.72	1.03	0.20	0.18	0.90	0.20	0.11	0.55	0.50	3.52	7.04
ps9	0.00	0.00	1.00	0.10	0.06	0.60	0.10	0.06	0.60	0.30	1.64	5.47
ps10	0.60	0.43	0.72	0.60	0.59	0.98	0.70	0.55	0.79	0.60	0.49	0.82
ps11	0.40	0.27	0.68	0.80	1.44	1.80	0.80	1.29	1.61	0.10	0.14	1.40
ps12	0.30	0.15	0.50	0.20	0.41	2.05	0.30	0.56	1.87	0.10	0.05	0.50

	Inflorescence			Shoot New Tip			Shoot 0.2m			Shoot 0.5m		
PS	Gene%	Exp.%	EP	Gene%	Exp.%	EP	Gene%	Exp.%	EP	Gene%	Exp.%	EP
ps1	38.40	32.14	0.84	35.50	35.31	0.99	31.90	27.25	0.85	31.70	31.89	1.01
ps2	28.40	24.41	0.86	43.70	39.68	0.91	34.50	33.42	0.97	37.70	33.91	0.90
ps3	13.30	18.21	1.37	4.80	3.71	0.77	9.90	9.54	0.96	7.30	6.50	0.89
ps4	11.00	10.00	0.91	8.80	7.34	0.83	15.50	14.88	0.96	13.50	13.07	0.97
ps5	2.80	4.11	1.47	1.40	1.25	0.89	1.60	1.58	0.99	1.30	1.11	0.85
ps6	3.30	3.32	1.01	3.10	9.01	2.91	4.30	10.66	2.48	5.20	10.33	1.99
ps7	0.70	1.69	2.41	0.30	0.31	1.03	0.50	0.40	0.80	0.30	0.17	0.57
ps8	0.50	4.16	8.32	0.20	0.12	0.60	0.60	0.39	0.65	0.70	0.97	1.39
ps9	0.20	0.52	2.60	0.10	0.04	0.40	0.10	0.09	0.90	0.10	0.05	0.50
ps10	0.70	0.76	1.09	0.80	0.90	1.13	0.50	0.33	0.66	0.20	0.12	0.60
ps11	0.20	0.13	0.65	0.90	1.44	1.60	0.60	1.46	2.43	0.90	1.07	1.19
ps12	0.50	0.55	1.10	0.40	0.89	2.23	0.00	0.00	1.00	1.10	0.80	0.73

	Shoot 1.0m			Shoot 2.0m			Shoot 3.0m			Shoot 5.0m		
PS	Gene%	Exp.%	EP	Gene%	Exp.%	EP	Gene%	Exp.%	EP	Gene%	Exp.%	EP
ps1	30.70	28.46	0.93	32.50	28.56	0.88	26.80	25.64	0.96	24.90	23.79	0.96
ps2	32.40	31.67	0.98	31.20	28.84	0.92	31.60	27.75	0.88	32.80	29.25	0.89
ps3	9.90	9.05	0.91	10.80	6.46	0.60	12.40	8.39	0.68	12.00	9.43	0.79
ps4	14.60	14.37	0.98	17.10	15.47	0.90	20.70	20.26	0.98	22.30	22.99	1.03
ps5	2.30	2.77	1.20	2.50	4.62	1.85	1.80	3.88	2.16	1.90	4.19	2.21
ps6	6.10	8.89	1.46	3.70	7.16	1.94	4.00	4.81	1.20	3.90	2.75	0.71
ps7	0.00	0.00	1.00	0.30	0.19	0.63	0.70	0.50	0.71	0.60	0.38	0.63
ps8	1.30	1.29	0.99	0.60	0.76	1.27	0.60	1.02	1.70	0.50	1.29	2.58
ps9	0.20	0.22	1.10	0.30	0.20	0.67	0.40	0.36	0.90	0.10	0.02	0.20
ps10	0.70	0.32	0.46	0.20	0.11	0.55	0.10	0.11	1.10	0.10	0.13	1.30
ps11	0.60	1.94	3.23	0.60	7.44	12.40	0.60	7.00	11.67	0.60	5.62	9.37

ps12	1.20	1.01	0.84	0.20	0.19	0.95	0.30	0.27	0.90	0.30	0.17	0.57

	Shoot 6.0m			Shoot 7.0m		
PS	Gene%	Exp.%	EP	Gene%	Exp.%	EP
ps1	25.00	24.82	0.99	25.40	26.63	1.05
ps2	32.60	29.32	0.90	32.40	27.26	0.84
ps3	13.70	11.38	0.83	13.50	11.49	0.85
ps4	20.70	20.84	1.01	20.20	19.82	0.98
ps5	2.20	4.20	1.91	2.20	5.53	2.51
ps6	3.90	2.89	0.74	3.80	3.06	0.81
ps7	0.20	0.16	0.80	0.30	0.21	0.70
ps8	0.50	1.18	2.36	0.70	1.01	1.44
ps9	0.00	0.00	1.00	0.10	0.03	0.30
ps10	0.20	0.16	0.80	0.30	0.18	0.60
ps11	0.60	4.91	8.18	0.80	4.66	5.83
ps12	0.40	0.14	0.35	0.30	0.12	0.40

Gene% represents the gene number of a given PS genes among 1 000 highly expressed genes.

Exp% represents the expression ration of a given PS genes among 1 000 highly expressed genes.

Supplementary Table 9. *De novo* genes identification in woody bamboos.

P. edulis	*B. amplexicaulis*	*O. latifolia*	*R. distichophylla*	*B. distachyon*	*O. sativa*
PH02Gene00431[0E]	scaffold47:5039209-5039650[0E]	scaffold268:306366-306730[SE]	GWHAAKD00038269:4795744-4795852[S]	NA	Chr4:33569992-33570157[S]
PH02Gene09097[0E]	scaffold1794:51699-52402[0E]	scaffold267:376983-379425[*SE]	GWHAAKD00038245:1848898-1849452[S]	NA	NA
PH02Gene09375[0E]	CCG041503[0]	scaffold232:499208-500241[SE]	GWHAAKD00035381:230880-231547[*S]	3:47744918-47745421[S]	NA
PH02Gene11274[0E]	CCG030975/CCG046696[0E]	scaffold238:10440-11096[*E]	GWHAAKD00028920:265915-266586[S]	4:44937841-44938310[S]	Chr12:4707026-4707390[S]
PH02Gene15427[0E]	CCG032424.2/CCG017348.2[0E]	scaffold81:1379087-1380416[*E]	NA	NA	NA
PH02Gene16241[0E]	CCG038660.1[0E]	scaffold806:30272-30455[S]	RdiGWHAAKD00028083:1403406-1403555[S]	NA	NA
PH02Gene17673[0E]	CCG036009.1[0E]	scaffold22:1928527-1929066[S]	NA	NA	Chr4:20698941-20699089[S]
PH02Gene20119[0E]	CCG041788.1[0E]	scaffold118:1086321-1086900[SE]	GWHAAKD00035697:1343150-1343726[S]	NA	Chr6:17812347-17812738[S]
PH02Gene28267[0E]	CCG027720.1[0]	scaffold274:1061056-1061629 [SE]	GWHAAKD00038218:132759-133311[S]	4:35644691-35645316[S]	Chr9:14608936-14609685[S]
PH02Gene28590[0E]	scaffold447:221869-227460[0E]	scaffold186:1174046-1174815 [SE]	GWHAAKD00038266:1348655-1349426[*S]	3:16622076-16622723[S]	NA
PH02Gene28800[0E]	CCG012322.1[0E]	scaffold231:196752-198501[*E]	NA	NA	NA
PH02Gene36478[0E]	CCG012322.1[0E]	scaffold231:196752-198501[*E]	NA	NA	NA
PH02Gene28893[0E]	CCG016091/CCG045817[0E]	scaffold95:1120070-1120871[SE]	GWHAAKD00038261:3857881-3858559[S]	NA	Chr10:16429102-16429793[S]
PH02Gene37270[0E]	CCG013122.1[0E]	scaffold130:376348-378691[*]	GWHAAKD00023579:1344352-1346251[*S]	1:30800404-30802632[*]	NA
PH02Gene38072[0E]	scaffold3:4267862-4272372[0E]	scaffold149:968644-970198[*]	GWHAAKD00017421:60619-62154[*]	NA	Chr1:10113276-10114161[S]
PH02Gene41079[0E]	scaffold1121:207703-208310[0E]	scaffold279:136882-137472[*]	GWHAAKD00032777:1739238-1739850[*]	3:2161811-2162396[S]	Chr2:1978677-1979266[S*]
PH02Gene43500[0E]	CCG021775.1[0]	scaffold491:275445-275583[S]	NA	NA	NA
PH02Gene43780[0E]	CCG007322.1[0]	scaffold491:275445-275583[S]	NA	NA	NA
PH02Gene43782[0E]	CCG007322.1[0]	scaffold491:275445-275583[S]	NA	NA	NA

Note: ‘0’ represents full ORF; ‘*’ represents premature stop codon;‘S’ represents a partial sequence (including large in-frame indels and incomplete gene structure (for example, an undetected start or stop codon or undetected splicing sites) ; NA represents absence homologous sequence; ‘E’ represents support by full-length transcriptome data in . *amplexicaulis* or in *O. latifolia*.

Supplementary Table 10. *De novo* genes with signals of natural selection resulting from the branch model analyses in PAML for orthologous gene pairs between *P. edulis* and *B. amplexicaulis*.

P. edulis gene	*B. amplexicaulis* gene	ω(Ka/Ks)	lnL(runmode=-2)	lnL(fix ω=1)	2ΔlnL	χ2 test p value (* p < 0.05)
PH02Gene17673	CCG036009	0.3791	-630.292308	-635.225611	9.866606	0.001683057*
PH02Gene38072	scaffold7188-3260-3685	0.2392	-300.430857	-302.048701	3.235688	0.072050036
PH02Gene09375	CCG041503	0.3266	-558.78786	-563.059144	8.542568	0.003469358*
PH02Gene28800	CCG012322	0.6506	-643.000039	-643.946184	1.89229	0.168943858
PH02Gene20119	CCG041788	0.0722	-1517.935673	-1533.724919	31.578492	0.000000019*
PH02Gene43780	scaffold1355-30690-30833	0.4843	-208.634932	-209.006616	0.743368	0.388584119
PH02Gene36478	CCG012322	0.5878	-577.560437	-578.493326	1.865778	0.171959415
PH02Gene16241	CCG038660	0.5707	-433.648994	-434.312705	1.327422	0.249264365
PH02Gene37270	CCG013122	1.443	-424.150497	-424.544357	0.78772	0.374790439
PH02Gene43500	CCG021775	0.2677	-251.20585	-254.605572	6.799444	0.009118627*
PH02Gene28590	CCG032132	0.0135	-1012.964538	-1030.125523	34.32197	0.000000005*
PH02Gene43782	CCG007322	0.4278	-554.725109	-558.291738	7.133258	0.007566712*
PH02Gene15427	CCG017348	0.4286	-1300.30583	-1307.90837	15.20508	0.000096444*
PH02Gene28893	CCG016090	5.0656	-479.274118	-481.257027	3.965818	0.046432957*
PH02Gene00431	CCG021938	0.019	-668.367811	-675.943009	15.150396	0.000099277*
PH02Gene11274	CCG030975	0.6746	-760.898747	-761.638494	1.479494	0.223853708
PH02Gene41079	scaffold1121-209400-210007	0.0673	-1243.286731	-1259.277559	31.981656	0.000000016*
PH02Gene09097	scaffold14:2096514-2097890	3.5425	-391.089967	-392.877249	3.574564	0.05867085
PH02Gene28267	scaffold743-2742347-2742847	99	-226.148466	-228.123781	3.95063	0.046853824*

Supplementary Table 11. The expression level of *de novo* genes.

De Novo genes	Root	Lateral bud	Rhizome	Leaf	Inflorescence	Shoot new tip	Shoot 0.2 m	Shoot 0.5 m	Shoot 1.0 m	Shoot 2.0 m	Shoot 3.0 m	Shoot 5.0 m	Shoot 6.0 m	Shoot 7.0 m
PH02Gene00431	0.108955	0.354002	0.439199	0.406334	0.227801	0.28961	0.875452	6.02932	0	0	0	0	1.1743	1.44279
PH02Gene28267	0	1.43058	2.0881	0.506803	0.678365	1.58723	2.44337	4.31871	2.63794	0.664708	0.81713	1.26964	1.18504	0.387471
PH02Gene37270	0	0.00345967	0.078956	0	0.112307	0.136841	0	4.5198	0	0	0	0	0	0
PH02Gene38072	0.768122	0.333078	0.154975	0.651582	0.371733	0.458837	0.566799	1.94744	0	0	0	0	0	0
PH02Gene41079	0.700928	0.0859407	0.348668	0.261332	0.261332	0.424032	0.123331	0.826558	6.17279	0	0	0	0	0
PH02Gene20119	0.532743	0.713629	0.545212	0.547303	0.989812	0.17824	0	0.466911	4.93642	0.0773267	0.330077	0.122076	0	0.266511
PH02Gene28590	20.4872	12.7251	10.0491	6.8659	4.124121	8.36692	11.1323	14.378	32.027	4.56044	2.51363	2.3923	5.36815	4.42987
PH02Gene15427	74.5786	126.562	145.83	0.0580967	8.087249	218.704	92.6947	97.2885	341.683	3300.57	3946.89	3101.16	3303.93	2382.59
PH02Gene17673	204.22	75.861	153.262	0.563891	15.779074	100.95	404.27	357.037	1143.27	9082.96	11336	6203.18	6774.16	4824.32
PH02Gene28800	110.118	296.631	215.981	49.9856	69.837723	251.133	354.909	156.432	487.049	2657.59	1889.96	1279.36	1373.72	970.495
PH02Gene36478	75.2958	685.432	513.3	1.39589	50.303837	596.555	986.134	885.132	1292.04	4783.04	3562.95	2331.86	2649.21	1950.07
PH02Gene09375	0.340232	0.088171	4.72408	0.0587473	0.2065	0.00802567	1.67809	0.125498	0	0	0	3.28017	15.4019	10.5318
PH02Gene11274	0.301629	0.0192843	0.09945	0.0902093	0.749388	0.021548	0	0.849924	0	0	0	0	0	0.546547
PH02Gene09097	10.9077	21.6762	12.4991	6.27018	11.730127	11.4153	4.17867	2.59689	2.3635	1.98299	4.91615	12.8236	4.28522	18.3584
PH02Gene16241	0.286101	12.4345	27.5881	0.0823797	0.015902	10.2779	6.75663	4.8989	15.9	1.27387	4.39143	3.08688	6.34196	4.45587
PH02Gene28893	28.5605	0.186734	0.0871793	4.56075	39.61335	0.0571693	0	0	0	0	0	0	0	0
PH02Gene43500	0.0886123	0	0	0.259672	1.065352	0	0	0	0	0	0	0	0	0
PH02Gene43780	0	0	0	7.83177	17.528885	0	0	0	0	0	0	0	0	0
PH02Gene43782	0	0	0	0.683648	1.89725	0	0	0	0	0	0	0	0	0.224359

Supplementary Table 12. *De novo* genes supported by proteomics data.

De novo genes	Peptide Modified Sequence	Shoots	Leaf/Seeding
PH02Gene28800	ASTEVETAPAVEAPAVEVPTPAEAVK	Y	N
PH02Gene28800	DTTAEATAEAEPAATLAPAADEPAVK	Y	N
PH02Gene28800	DTTAEATAEAEPAATLAPAADEPAVKEDPPVEEK	Y	N
PH02Gene28800	EDPPVEEK	Y	N
PH02Gene28800	TKDTTAEATAEAEPAATLAPAADEPAVK	Y	N
PH02Gene28800	TKDTTAEATAEAEPAATLAPAADEPAVKEDPPVEEK	Y	N
PH02Gene28800	TPEAEPEVTEPPAEPTEK	Y	Y
PH02Gene17673	EPEVGAK	Y	N
PH02Gene17673	EQEPAAAEAETK	Y	Y
PH02Gene15427	EAEPTAAEAEPVPAEAEAK	Y	N
PH02Gene15427	EAEPTAAEAEPVPAEAEAKEPAEAVPTDEAK	Y	N
PH02Gene15427	EPAEAVPTDEAK	Y	N
PH02Gene15427	GAEPEAAPAETEPEAAPVETETKEPEAETK	Y	N
PH02Gene36478	AEDTPAAEAPADAEPAAAPAPAADEPAVK	Y	N
PH02Gene36478	AEDTPAAEAPADAEPAAAPAPAADEPAVKEDPPVEEK	Y	N
PH02Gene36478	APEDEPEVTEPPAEPTEK	Y	N
PH02Gene36478	ASTEVATAPAVEAPAVEVPAVETPAEVVK	Y	N
PH02Gene36478	EDPPVEEK	Y	N
PH02Gene36478	EDPPVEEKAPEDEPEVTEPPAEPTEK	Y	N

Supplementary Table 13. Genic characteristics of *Phyllostachys edulis* genes in each phylostratum.

PS	CDS length	Exon number	*Ka/Ks*	*Tau*
PS1	1350 (1179) (150 - 16209)	6.6 (5.0) (1 - 65)	0.26 (0.21) (0.00 – 8.71)	0.4473 (0.4690)
PS2	1303 (1050) (150 - 16407)	6.4 (5.0) (1 - 78)	0.31 (0.25) (0.00 – 5.1)	0.4216 (0.4230)
PS3	1044 (897) (154 - 8361)	4.7 (4.0) (1 - 33)	0.31 (0.25) (0 - 8.5)	0.4953 (0.5380)
PS4	1054 (891) (153 - 8031)	3.9 (3.0) (1 -28)	0.32 (0.24) (0 – 11.07)	0.5041 (0.5810)
PS5	927.7 (712.5) (160 - 4614)	3.4 (3.0) (1 - 21)	0.34 (0.26) (0 – 2.68)	0.5321 (0.6285)
PS6	908.3 (672.0) (192 - 12039)	3.0 (2.0) (1 - 23)	0.42 (0.31) (0 – 5.16)	0.5602 (0.6830)
PS7	582.4 (480.0) (162 - 2553)	2.9 (2.0) (1 - 17)	0.58 (0.40) (0.01 – 3.73)	0.6020 (0.7800)
PS8	615.4 (513.0) (177 - 2424)	2.8 (2.0) (1 - 16)	0.65 (0.45) (0 – 8.43)	0.6609 (0.8380)
PS9	495.4 (411) (164 - 2325)	3.2 (3.0) (1 - 13)	1.10 (0.89) (0 – 5.34)	0.5930 (0.7410)
PS10	429.1 (336.5) (151 - 3406)	3.4 (3.0) (1 - 9)	1.42 (0.94) (0 – 12.23)	0.5517 (0.8310)
PS11	371.5 (309.0) (150 - 1502)	3.0 (3.0) (1 - 8)	1.21 (0.89) (0 – 9.06)	0.6288 (0.8240)
PS12	355.1 (303.0) (150 - 1957)	3.2 (3.0) (1 - 15)	1.32 (0.92) (0 – 6.90)	0.6412 (0.8320)

Average (median) (min - max)

Supplementary Table 14. Specifically expressed genes (SEGs) of seven tissues in *P. edulis* tissues.

Tissues	Root	Lateral bud	Leaf	Rhizome tip	Inflorescences	Shoot tip	Shoot								
							0.2m	0.5m	1.0m	2.0m	3.0m	5.0m	6.0m	7.0m	0.2-7.0m
PS1	163	10	143	3	654	10	19	543	490	5	5	0	0	2	325
PS2	135	11	88	3	48	15	30	724	653	8	3	0	4	2	369
PS3	81	9	71	4	23	3	12	198	214	3	2	0	7	2	171
PS4	120	10	47	7	45	8	25	379	342	3	4	0	1	3	292
PS5	16	2	0	1	0	1	5	49	39	1	0	0	0	0	44
PS6	36	1	0	4	0	1	7	147	134	0	3	0	0	0	98
PS7	6	0	0	0	0	0	1	13	15	0	0	0	0	1	13
PS8	7	0	0	0	0	1	0	46	45	0	0	0	0	1	31
PS9	2	0	1	0	0	0	0	11	5	0	0	0	0	0	8
PS10	2	0	1	0	2	0	0	13	13	0	0	0	0	0	9
PS11	2	0	1	0	3	2	0	18	13	0	0	0	0	0	5
PS12	4	0	4	0	8	1	1	63	60	0	0	0	0	0	30
Total							100	2204	2023	20	17	0	12	11	1395
	574	43	356	22	194	42					5782				

Supplementary Table 17. Specifically expressed genes (SEGs) of four tissues in rice

Gene age	Seeds			Leave	Inflorescences			Shoots
	5 & 10 days	Embryo	Endosperm		Pre- & Post-emerged	Pistil	Anther	
PS1	30	64	15	69	110	26	213	199
PS2	41	39	22	44	122	57	294	121
PS3	26	26	10	40	44	22	72	71
PS4	48	54	23	41	116	43	164	98
PS5	9	8	1	4	17	7	24	12
PS6	24	31	12	27	55	27	88	47
PS7	1	6	2	2	8	2	13	6
PS8	16	6	15	6	38	8	63	25
PS9	4	2	1	2	5	5	10	9
PS10	6	6	1	1	4	4	9	5
PS11	4	34	5	14	11	13	59	24
PS12	2	4	0	1	2	3	10	8
Total		598		251		1768		625

Supplementary Table 19. Thirteen co-expression modules were identified in the WGCNA analysis.

**Modules**	**PS1**	**PS2**	**PS3**	**PS4**	**PS5**	**PS6**	**PS7**	**PS8**	**PS9**	**PS10**	**PS11**	**PS12**	**Total**
Turquoise	1147	1817	302	496	51	145	12	19	5	17	8	22	4041
Blue	582	776	219	455	38	115	15	17	3	5	5	14	2244
Brown	654	391	294	205	11	36	2	18	6	8	2	5	1632
Green	207	258	109	174	16	42	4	10	1	3	3	10	837
Purple	48	78	12	36	7	16	0	1	1	0	0	3	202
Black	127	134	58	110	9	32	6	0	4	4	4	4	492
Red	244	197	115	187	16	38	7	9	3	5	2	6	829
Greenyellow	54	48	13	42	9	13	3	2	4	0	1	3	192
Tan	32	51	32	38	3	8	1	2	0	0	1	1	169
Pink	241	157	25	38	4	7	0	1	0	1	0	1	475
Yellow	324	397	119	181	30	65	0	8	1	2	1	2	1130
Magenta	211	104	66	41	4	12	1	2	0	2	1	2	446
Grey	11	15	3	6	2	2	0	0	0	0	0	0	39
Total	3882	4423	1367	2009	200	531	51	89	28	47	28	73	12728

Supplementary Table 20. The top 50 connected genes of *de novo* gene *PH02Gene28800* according to the weight value of black module in the WGCNA analysis.

GO ID	GO term description	Connection Node	Prefername name	SBW
GO:0071554	cell wall organization or biogenesis	PH02Gene23275.t1	GXM3	Yes
GO:0071554	cell wall organization or biogenesis	PH02Gene21323.t1	XTH30	No
GO:0071554	cell wall organization or biogenesis	PH02Gene30719.t1	GXM3	Yes
GO:0071554	cell wall organization or biogenesis	PH02Gene21702.t1	IRX6	No
GO:0071554	cell wall organization or biogenesis	PH02Gene35057.t1	CESA6	No
GO:0071554	cell wall organization or biogenesis	PH02Gene45992.t1	CESA8	No
GO:0071554	cell wall organization or biogenesis	PH02Gene34577.t1	CESA8	No
GO:0071554	cell wall organization or biogenesis	PH02Gene21681.t1	UGD1	Yes
GO:0071554	cell wall organization or biogenesis	PH02Gene21682.t1	UGD1	No
GO:0071554	cell wall organization or biogenesis	PH02Gene13564.t1	HCT	No
GO:0009725	response to hormone	PH02Gene21671.t1	EFE	Yes
GO:0009725	response to hormone	PH02Gene04302.t1	MYB61	No
GO:0009725	response to hormone	PH02Gene04901.t1	PIN1	No
GO:0009725	response to hormone	PH02Gene36937.t1	AMP1	No
GO:0009725	response to hormone	PH02Gene26386.t1	PIP3	No
GO:0009725	response to hormone	PH02Gene04108.t1	ASPG1	No
GO:0009725	response to hormone	PH02Gene43754.t1	PORA	Yes
GO:0009725	response to hormone	PH02Gene13991.t1	BT1	No
GO:1901657	glycosyl compound metabolic process	PH02Gene43588.t1	HOG1	Yes
GO:1901657	glycosyl compound metabolic process	PH02Gene33354.t1	CDA1	No
GO:1901657	glycosyl compound metabolic process	PH02Gene24548.t1	No	No
GO:1901657	glycosyl compound metabolic process	PH02Gene40439.t4	ADK2	No
GO:0005975	carbohydrate metabolic process	PH02Gene46034.t1	SUS1	No
GO:0005975	carbohydrate metabolic process	PH02Gene00159.t1	UXS3	Yes
GO:0040008	regulation of growth	PH02Gene15974.t1	bHLH	No
GO:0040008	regulation of growth	PH02Gene44483.t1	No	No
GO:0006807	nitrogen compound metabolic process	PH02Gene02354.t1	MC5	No
GO:0006807	nitrogen compound metabolic process	PH02Gene11009.t1	**ATL2**	No
GO:0006807	nitrogen compound metabolic process	PH02Gene31547.t1	PGDH	Yes
GO:0006807	nitrogen compound metabolic process	PH02Gene11761.t1	RKL1	Yes
GO:0006807	nitrogen compound metabolic process	PH02Gene23184.t1	BAG1_a	No
GO:0006807	nitrogen compound metabolic process	PH02Gene26208.t1	BAG1_b	No
GO:0071840	cellular component organization or biogenesis	PH02Gene17884.t1	TUA3	No
GO:0071841	cellular component organization or biogenesis	PH02Gene00173.t1	No	No
No	No	PH02Gene28292.t1	No	No
No	No	PH02Gene44012.t1	No	No
No	No	PH02Gene35620.t1	EMP1	No
No	No	PH02Gene40632.t1	No	No

No	No	PH02Gene11671.t1	No	No
No	No	PH02Gene31299.t1	No	No
No	No	PH02Gene25401.t1	No	No
No	No	PH02Gene47897.t1	No	No
No	No	PH02Gene31078.t2	No	No
No	No	PH02Gene04257.t1	FLA6	No
No	No	PH02Gene07522.t4	No	No
No	No	PH02Gene16888.t1	No	No
No	No	PH02Gene47866.t1	No	No
No	No	PH02Gene46868.t1	VAP27-1	No
No	No	PH02Gene18072.t1	ENoDL17	Yes
No	No	PH02Gene41745.t1	LBD11	No

Supplementary Table 21. Summary of PacBio single-molecule long-read sequencing

Species	*Bonia amplexicaulis*	*Olyra latifolia*
Samples	leaves and shoots	leaves and stems
Data size	21.38 Gb	25.52 Gb
cDNA Size	1-6K	1-6K
Number of CCS	535 064	406 686
Number of full-length Non-chimeric reads (FLNCs) (%)	421 784 (78.83%)	362 629 (89.17%)
Cluster	187 511	227 940
Non-redundant	107 598	73 457

Supplementary Table 22. Comparison of phylostratigraphic analysis of *Phyllostachys edulis* before and after adding PacBio full length transcriptomic data.

Phylostratum	Lineage	Set 1	Set 2
ps1	Archaea & Bacteria	5857	5857
ps2	Eukaryotes	8117	8117
ps3	Green algae	2982	2982
ps4	Bryophytes	5194	5194
ps5	Lycophytes	603	603
ps6	Angiosperm	2267	2267
ps7	MoNocots	256	256
ps8	Poaceae	764	764
ps9	BOP clade	172	172
ps10	Bambusoideae	**45**	**362**
ps11	Woody bamboo	**97**	**234**
ps12	*Phyllostachys edulis*	**1439**	**985**
Total		27793	27793

Note: Set 1 represents reference data without full length transcriptome data. Set 2 represents reference data with full length transcriptome data.
